# Electronic Properties of a Structural Model of Single-Atom Co-Adsorption to MoS**_2_** Edge Sites

**DOI:** 10.1021/acs.inorgchem.6c01985

**Published:** 2026-06-03

**Authors:** Leyla R. Valerio, Isabella Florez Monroy, Zhou Lu, William W. Brennessel, Ellen M. Matson

**Affiliations:** Department of Chemistry, 6927 University of Rochester, Rochester, New York 14627, United States

## Abstract

Molecular-level insight into how single-atom transition metal dopants modulate electronic structure at the edge sites of MoS_2_ remains an underdeveloped area of research that is essential for the design of efficient catalysts. The present work describes the synthesis of a cobalt-substituted thiomolybdate dimer, [(CpMo)_2_(Cp*Co)­(μ_3_-S)_2_(μ_2_-S_2_CH_2_)], which serves as a molecular model for cobalt adsorption at the MoS_2_ edge. Spectroscopic and magnetic measurements establish a diamagnetic, closed-shell ground state for the heterobimetallic complex. Time-dependent density functional theory (TD-DFT) calculations indicate significant electronic communication across the “CoMo_2_S_4_” assembly. Electrochemical studies reveal three reversible redox processes, assigned to a Co-centered reduction and Mo-based oxidations. Access to the reduced and oxidized species was possible, allowing for investigation into redox-dependent changes to electronic structure. Reduction centered at the cobalt atom generates a Co­(II)­Mo­(III)_2_ complex, whereas one-electron oxidation affords a mixed-valent Co­(III)­Mo­(IV)­Mo­(III) species that features electron delocalization across the “Mo_2_” subunit. Our findings demonstrate that cobalt incorporation significantly impacts the charge distribution and magnetic properties of the thiomolybdate, providing insight into the effects of transition metal uptake at MoS_2_ edge sites.

## Graphical abstract

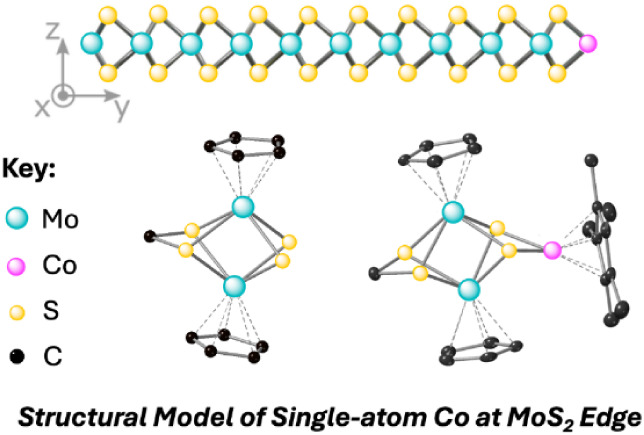

## Introduction

Single-atom catalysts (SACs) have emerged as a promising class of electrocatalysts for a variety of energy conversion processes due to their ability to maximize atom efficiency and provide well-defined active sites.^1–5^ These atomically dispersed catalysts consist of isolated metal atoms or mononuclear metal complexes anchored on a substrate, where their structural stability and catalytic activity often rely on the local chemical environment and intrinsic properties of the adsorbed metal atom.^6–8^ Consequently, electronic communication between the redox-active support and the adsorbed transition metal ion plays a critical role in determining the performance of SACs.^6,9,10^

Among redox-active supports that have been investigated, layered molybdenum disulfide (MoS_2_) has received considerable attention because of its abundance, low cost, and tunable electronic properties.^11–15^ However, the catalytic activity of MoS_2_ is localized at the sulfur-rich edges, where disulfide linkages or triangular units are exposed, rendering much of the material inert.^16–19^ This limitation has motivated experimental and theoretical efforts to introduce single metal atoms at the basal plane and edge sites of MoS_2_ to activate the surface for catalytic transformations, including hydrogen evolution, CO_2_ reduction, and hydrodeoxygenation.^2,6,20–22^ These studies have demonstrated that the installation of single-atom dopants on the surface of MoS_2_ can significantly alter its electronic structure, enhancing the catalytic performance of the material. Despite these advances, most studies rely on heterogeneous materials, which can result in a lack of precise understanding of the interactions between single-atom dopants and MoS_2_ that dictate reactivity at the molecular level.

To address this challenge, molecular models have been developed to provide well-defined representations of MoS_2_ active site motifs (Figure 1). These discrete complexes enable systematic investigation of structure–function relationships while offering atomistic insight into electronic structure and reactivity. In this context, cuboidal trinuclear thiomolybdate clusters of the general formula L_3_Mo_3_S_4_ have emerged as useful models for the basal plane of MoS_2_.^23–25^ For example, Ohki and coworkers demonstrated that Cp*_3_Mo_3_S_4_ (Cp* = 1,2,3,4,5-pentamethylcyclopentadienyl) could be functionalized with cobalt to generate (Cp*_3_Mo_3_S_4_)­CoCl, representing a molecular analogue of single-atom Co-adsorption at the basal plane of MoS_2_, though we note that reactivity studies of this cluster remain limited.^26^ Molecular models of MoS_2_ edge sites have likewise provided important insight into the origins of catalytic activity.^27,28^ DuBois and coworkers have demonstrated the utility of the thiomolybdate dimer, (CpMo­(μ-S))_2_S_2_CH_2_ (Cp = cyclopentadienyl), as a representative edge-site model of MoS_2_, showing that it serves as a highly selective catalyst for the electrochemical reduction of protons to hydrogen with a near-quantitative current efficiency.^27^ Given the increased nucleophilicity and catalytic relevance of these edge sites, understanding the impact of single-atom transition metal uptake at this facet of MoS_2_ is an important area for establishing design principles that dictate how dispersed single atoms tune the electronic structure of different facets of MoS_2_.

**Figure 1:**
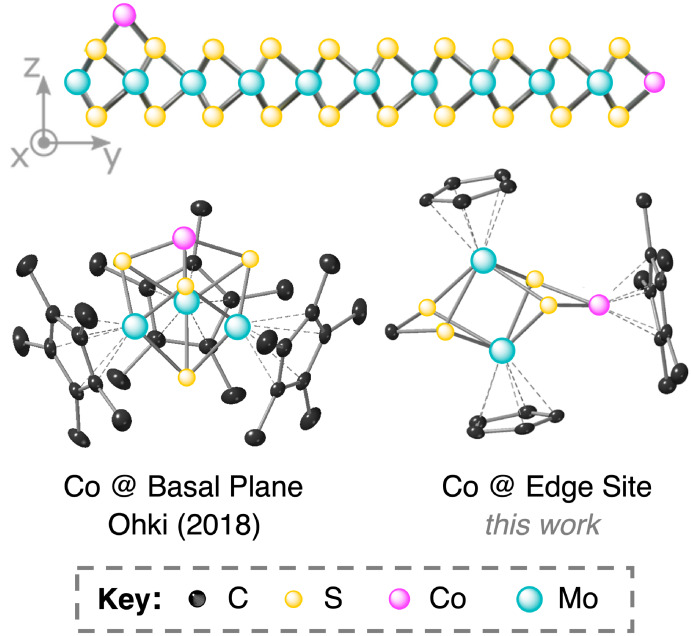
Selected examples of molecular models for cobalt doping at different facets of MoS_2_.

Our group has been interested in exploring the synthesis of cobalt-substituted thiomolybdate clusters as structural and functional models for Co-doped MoS_2_. In recent work, we demonstrated that Cp*_3_CoMo_2_S_4_ functions as a homogeneous catalyst for the hydrogen evolution reaction (HER).^29^ Comparative studies with the all-molybdenum analogue of the cluster, Cp*_3_Mo_3_S_4_, reveal improved catalytic performance toward proton reduction upon cobalt incorporation. Notably, Cp*_3_CoMo_2_S_4_ serves as a molecular model for cobalt substitution within the basal plane of MoS_2_, whereas (Cp*_3_Mo_3_S_4_)­CoCl represents a model of cobalt adsorption on the surface.^26^ Building on these findings, we became interested in extending this series to the edge sites of MoS_2_ to investigate how cobalt incorporation influences the electronic structure and reactivity of the thiomolybdate framework at this position.

Herein, we employ a dimeric thiomolybdate platform with a tethered backbone, (CpMo­(μ-S))_2_S_2_CH_2_, as a molecular model for the edge sites of MoS_2_.^27^ The tether serves to block one face of the dimer, enforcing single-site binding of transition metals. Incorporation of a Cp*Co fragment enables interrogation of Co adsorption at the sulfur-rich edge site (Figure 1). We hypothesized that incorporation of cobalt would modulate the electronic structure of the thiomolybdate assembly and, in turn, its reactivity. Through combined spectroscopic, electrochemical, and computational studies, we demonstrate that cobalt incorporation significantly perturbs the electronic structure of the thiomolybdate assembly, allowing for isolation of three redox states of the complex, [(CpMo)_2_(Cp*Co)­(μ_3_-S)_2_(μ_2_-S_2_CH_2_)]^n^ (n = 1-, 0, 1+). Each redox state possesses distinct electronic structures, highlighting the changes in metal–metal communication as a function of the charge state of the assembly.

## Experimental Section

### General Considerations

All air- and moisture-sensitive manipulations were carried out using standard high-vacuum line, Schlenk, or cannula techniques, or in an MBraun inert atmosphere drybox containing an atmosphere of purified dinitrogen. Solvents for air- and moisture-sensitive manipulations were dried and deoxygenated using a Glass Contour Solvent Purification System (Pure Process Technology, LLC) and stored over activated 4 Å molecular sieves (Fisher Scientific) prior to use. Deuterated solvents for ^1^H NMR spectroscopy were purchased from Cambridge Isotope Laboratories and stored in the glovebox over activated 3 Å molecular sieves after three freeze–pump–thaw cycles. [(CpMo)_2_(μ_2_-S)_2_(μ_2_-S_2_CH_2_)],^30^ KC_8_,^31^ and (Cp*CoCl)_2_ ^32^ were synthesized following established literature procedures (see relevant references).

#### Synthesis of [(CpMo)_2_(Cp*Co)­(μ_3_-S)_2_(μ_2_-S_2_CH_2_)] (**1**)

A 20 mL scintillation vial was charged with [(CpMo)_2_(μ_2_-S)_2_(μ_2_-S_2_CH_2_)] (0.040 g, 0.086 mmol) and 5 mL of tetrahydrofuran (THF). The solution was placed in the drybox freezer for 10 min to cool to −30 °C and was subsequently added to a vial containing solid potassium graphite (KC_8_; 0.012 g, 0.086 mmol, 1 equiv). The mixture was stirred for 30 s before a solution of (Cp*CoCl)_2_ (0.020 g, 0.043 mmol, 0.5 equiv) in 5 mL of THF at −30 °C was added. After stirring for 1 h at room temperature, the reaction mixture was filtered over Celite (1 cm). Volatiles were removed from the filtrate under reduced pressure. The remaining solid was extracted into toluene, filtered again over Celite (1 cm), and dried under reduced pressure. The product was washed with ∼2 mL of acetonitrile and dried *in vacuo* to afford compound **1** as a dark blue powder. Yield: 0.04 g, 0.061 mmol, 70%. Blue crystals suitable for single-crystal X-ray diffraction were grown from a concentrated solution of the product in *N,N-*dimethylformamide (DMF) at room temperature. ^1^H NMR (400 MHz, C_6_D_6_) δ = 5.67 (s, 10H), 4.94 (s, 2H), 1.71 (s, 15H). Electronic absorption spectrum: λ = 340 nm (ε = 29,519 M^–1^ cm^–1^), 614 nm (5,742 M^–1^ cm^–1^), and 955 nm (2,513 M^–1^ cm^–1^). Anal. Calcd for C_21_H_27_CoMo_2_S_4_ (mol.wt. 658.409 g/mol): C, 40.09%; H, 4.29%; N, 0%. Found: C, 40.26%; H, 4.09%; N, −0.10%.

#### Synthesis of [K­(2.2.2-cryptand)]­[(CpMo)_2_(Cp*Co)­(μ_3_-S)_2_(μ_2_-S_2_CH_2_)] (**2**)

A 20 mL scintillation vial was charged with **1** (0.015 g, 0.023 mmol) and 5 mL of tetrahydrofuran (THF). The solution was then added to a vial of KC_8_ (0.004 g, 0.023 mmol, 1 equiv), resulting in a color change to dark brown. The mixture was stirred for 30 s. A solution of 2.2.2-cryptand (0.009 g, 0.023 mmol, 1 equiv) in 1 mL of THF was added to the previous vial dropwise and stirred for an additional 10 min. The reaction mixture was filtered over Celite (1 cm), and the solvent was removed under reduced pressure. The product was washed with pentane and dried *in vacuo* to afford the title compound. Yield: 0.015 g, 0.014 mmol, 90%. Green-brown crystals suitable for single-crystal X-ray diffraction were grown from the slow diffusion of pentane into a concentrated THF solution of the product at −30 °C. ^1^H NMR (500 MHz, THF) δ = 5.60 (s, 10H), 3.67 (m, 36H), 2.65 (s, 15H). Magnetic moment: μ_eff_ = 1.75 μ_B_ (Evans method, ^1^H NMR, THF-*d*_8_, 25 °C). Electronic absorption spectrum: λ = 340 nm (ε = 12,134 M^–1^ cm^–1^), 606 nm (ε = 1,031 M^–1^ cm^–1^), and 948 (ε = 281 M^–1^ cm^–1^). Anal. Calcd for C_39_H_63_KCoMo_2_N_2_O_6_S_4_ (mol.wt. 1074.113 g/mol): C, 43.61%; H, 5.91%; N, 2.61%. Found: C, 43.61%: H, 6.18%; N, 2.75%.

#### Synthesis of [(CpMo)_2_(Cp*Co)­(μ_3_-S)_2_(μ_2_-S_2_CH_2_)]­[PF_6_] (**3**)

A 20 mL scintillation vial equipped with a stir bar was charged with **1** (0.015 g, 0.023 mmol) and 3 mL of dichloromethane (DCM). One equivalent of ferrocenium hexafluorophosphate (FcPF_6_; 0.008 g, 0.024 mmol) dissolved in 2 mL of DCM was added to the stirring solution, and the resulting mixture was allowed to stir for 5 min. The reaction mixture was filtered over Celite with a glass microfiber plug, and the solvent was removed under reduced pressure. The product was washed with pentane and dried *in vacuo* to afford the title compound. Yield: 0.015 g, 0.019 mmol, 83%. Brown crystals suitable for single-crystal X-ray diffraction were grown from the slow diffusion of diethyl ether into a concentrated DCM solution of the product at −30 °C. ^1^H NMR (500 MHz, CD_3_CN) δ 8.15 (br, s). μ_eff_ = 3.85 μ_B_ (Evans method, ^1^H NMR, CD_3_CN, 25 °C). Electronic Absorption: λ = 400 nm (ε = 3,031 M^–1^ cm^–1^), 529 nm (ε = 1,257 M^–1^ cm^–1^), 664 (ε = 984 M^–1^ cm^–1^), and 780 (ε = 1,047 M^–1^ cm^–1^). Anal. Calcd for C_21_H_27_CoMo_2_S_4_PF_6_ (mol.wt. 803.484 g/mol): C, 29.74%; H, 3.29%; N, 0%. Found: C, 29.30%: H, 3.06%; N, 0.23%.

### Physical Measurements

^1^H NMR spectra were recorded at room temperature on a 400 MHz Bruker AVANCE spectrometer or a 500 MHz Bruker AVANCE spectrometer locked on the signal of deuterated solvents. All chemical shifts are reported relative to the chosen deuterated solvent as a standard. Solution-phase effective magnetic moments were determined using the Evans method by ^1^H NMR spectroscopy. A dried solid analyte sample was dissolved in 0.600 μL of deuterated solvent and placed in an NMR tube equipped with a capillary containing the same solvent. Spectra were collected at room temperature on a 400 or 500 MHz Bruker AVANCE spectrometer. The frequency shift between the solvent resonance in the sample and the pure reference solvent was used to determine magnetic susceptibility. The frequency difference between the reference and sample (Δ*υ*, measured in Hz) was used to calculate the molar magnetic susceptibility (*χ_M_*) using the following equation: $\chi_{M}=\left(\frac{3\Delta \nu }{4\pi \nu_{0}C}+\chi_{dia}\right)MW$ , where *υ*_0_ is the spectrometer frequency in MHz, *C* is the concentration of the paramagnetic compound in solution, *χ_dia_* is the diamagnetic correction for organic ligands, and *MW* is the molecular weight of the compound. Effective magnetic moments (μ_eff_) were calculated using the following equation: μ_eff_ = $2.828\sqrt{\chi_{M}T}$ . X-band electron paramagnetic resonance (EPR) measurements were carried out on a Bruker EMXplus spectrometer (microwave frequency of 9.382 GHz) at 10 K. Electronic absorption measurements were recorded at room temperature in dimethylformamide (DMF) solution in sealed 1 cm quartz cuvettes using an Agilent Cary 6000i UV–vis/NIR spectrophotometer. Molar absorptivity values were determined using the following equation: ε = A/cL, where *A* = the absorbance value at a specific wavelength, *c* = the concentration of the sample, and *L* = the path length of the cuvette (1 cm). Elemental analysis data were obtained from the Elemental Analysis Facility at the University of Rochester. Microanalysis samples were weighed with a PerkinElmer model AD6000 autobalance, and their compositions were determined with a PerkinElmer 2400 Series II analyzer. Air-sensitive samples were handled in a VAC Atmospheres glovebox.

Cyclic voltammetry experiments were performed using a three-electrode setup inside a nitrogen-filled glovebox (MBraun UniLab, USA) using a Bio-Logic SP 150 potentiostat/galvanostat and the EC-Lab software suite. The concentration of the analyte and the supporting electrolyte (tetrabutylammonium hexafluorophosphate, TBAPF_6_) was 1 mM and 100 mM, respectively, across all measurements. Cyclic voltammograms (CVs) were recorded using a 3 mm diameter glassy carbon working electrode (CH Instruments, USA), a Pt wire auxiliary electrode (CH Instruments, USA), and an Ag wire reference electrode (CH Instruments, USA). Ferrocene (Fc) was added as an internal standard after the completion of the measurements, and all potentials were referenced versus the Fc^+^/Fc^0^ couple. CVs were IR-compensated at 85% with impedance taken at 100 kHz using the ZIR tool included within the EC-Lab software. Bulk electrolysis experiments were performed in an H-cell with a glass frit separator (porosity = 10–16 μm, Pine Research, USA) using a Bio-Logic SP 150 potentiostat/galvanostat. An active species concentration of 0.01 M was used. The working electrode compartment contained 5 mL of the active species with 0.1 M supporting electrolyte TBAPF_6_ in the desired solvent (DMF), while the counter electrode compartment had 5 mL of 0.1 M supporting electrolyte in the same solvent. A Pt mesh working electrode and a Pt wire counter electrode were used. Bulk electrolysis experiments were carried out using the chronoamperometry techniques available in the EC-Lab software suite at constant potentials selected from CV.

### X-ray Crystallography

In separate experiments, single crystals of **1**, **2**, and **3** were placed on a nylon loop and mounted on a Rigaku XtaLAB Synergy-S Dualflex diffractometer equipped with a HyPix-6000HE HPC area detector for data collection at 100.00(10) K. A preliminary set of cell constants and an orientation matrix were calculated from a small sampling of reflections. A short pre-experiment was run, with both CuKα and MoKα radiation, from which an optimal data collection strategy was determined. All data for the reported crystal structures were ultimately collected with CuKα radiation, as the results of the pre-experiments indicated that using MoKα radiation did not offer any significant improvement in structure quality but greatly increased the collection time. After the intensity data were corrected for absorption, the final cell constants were calculated from the xyz centroids of the strong reflections from the actual data collections after integration. The structure was solved using SHELXT^33^ and refined using SHELXL.^34^ Most or all non-hydrogen atoms were assigned from the solution. Full-matrix least-squares/difference Fourier cycles were performed, which located any remaining non-hydrogen atoms. All of the non-hydrogen atoms were refined with anisotropic displacement parameters. All of the hydrogen atoms were placed in ideal positions and refined as riding atoms with relative isotropic displacement parameters. For more details on the particulars of the X-ray diffraction experiments, we refer readers to the Electronic Supporting Information file associated with this work.

### Computational Methods

All calculations were conducted using the ORCA 6.0.1 software package.^35^ The geometry optimization adopted the crystal structure as an initial guess and was performed under the TPSSh-D4 level of theory using Def2-TZVP as a basis set.^36^ No imaginary frequencies were found to ensure the minima. Time-dependent density functional theory (TD-DFT) calculations were performed using the double-hybrid revDSD-PBEP86-D4(2021) functional^37^ with the consideration of the SMD solvation model^38^ of DMF. Electronic excitation and electron density difference (EDD) maps were analyzed and generated by Multiwfn 3.8;^39^ all of the EDD maps were plotted with an isovalue of 0.0007 au.

## Results and Discussion

### Synthesis and Characterization of [(CpMo)_2_(Cp*Co)­(μ_3_-S)_2_(μ_2_-S_2_CH_2_)]

Building on our prior work examining how cobalt dopants influence the electronic structure and reactivity of thiomolybdate clusters,^29^ we sought to develop molecular model systems for MoS_2_ edge sites with adsorbed cobalt atoms. The dimeric thiomolybdate assembly, [(CpMo)_2_(μ_2_-S)_2_(μ_2_-S_2_CH_2_)],^27,30^ originally reported by DuBois and coworkers as an active electrocatalyst for proton reduction, was chosen as a representative model of MoS_2_ edges. Notably, the organic tether (i.e., methylene bridge) on one face of the assembly blocks interactions between transition metals and the opposite face of the dimer, restricting reactivity to the bridging sulfide ligands. This structural feature provides opportunities for the selective uptake of single transition metal atoms, providing a good model for investigating the local electronic properties resulting from the deposition of single-atom transition metals at the edge sites of MoS_2_.

Addition of half an equivalent of (Cp*CoCl)_2_ to [(CpMo)_2_(μ_2_-S)_2_(μ_2_-S_2_CH_2_)] in the presence of a reductant results in the isolation of the target complex, [(CpMo)_2_(Cp*Co)­(μ_3_-S)_2_(μ_2_-S_2_CH_2_)] (**1**) (Scheme 1; see Experimental Section for additional details pertaining to the isolation of this compound). Following workup, the dark blue-green product was characterized via ^1^H NMR spectroscopy. The spectrum reveals complete consumption of the starting materials, with three new resonances observed at 5.67 (10H), 4.93 (2H), and 1.72 (15H) ppm, assigned to the Cp–H protons bound to Mo, the CH_2_ protons of the bridging methanedithiolate ligand, and the Cp*–H protons bound to Co, respectively (Figure S1). Product formulation is further supported by elemental analysis (see Experimental Section).

**Scheme 1:**
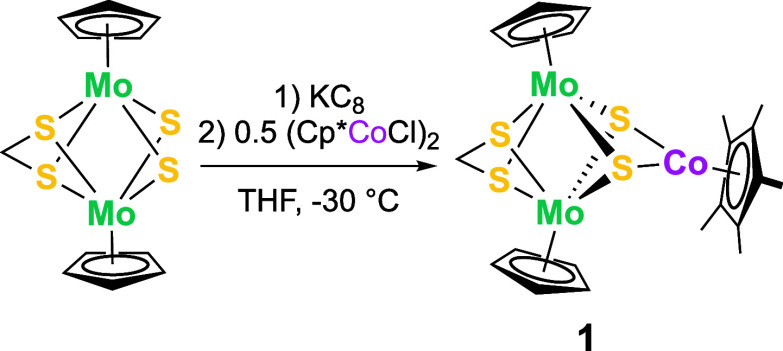
Synthesis of [(CpMo)_2_(Cp*Co)­(μ_3_-S)_2_(μ_2_-S_2_CH_2_)] (**1**)

Confirmation of product formation was obtained through single-crystal X-ray diffraction (SCXRD). Refinement of the data reveals coordination of cobalt to the bridging sulfide ligands of the thiomolybdate dimer, with an η^5^-Cp* ligand completing the coordination sphere of the heterometal (Figure 2, Tables 1 and S1). The observed coordination environment of cobalt matches that predicted by density functional theory (DFT) calculations for the binding of metal atoms at the S-edge of MoS_2_.^40,41^ Interestingly, the solid-state structure reveals that the cobalt dopant is tilted toward Mo(1), with a Co–Mo(1) distance of 2.8291(4) Å. By comparison, the Co–Mo(2) distance is much longer (3.4589(6) Å), suggesting a potential interaction between the Co and Mo(1) metal centers in the solid state. Indeed, the Co–Mo(1) bond distance is only slightly elongated from prior reports of other Co–Mo single bonds, which range from 2.618(8)–2.806(7) Å.^42–46^ Evidence for an interaction between Co and Mo(1) is also observed in the Mo–S–Co bond angles, where the Mo(1)–S–Co angle is 76.15(2)° and the Mo(2)–S–Co angle is 97.21(3)°.

**Figure 2:**
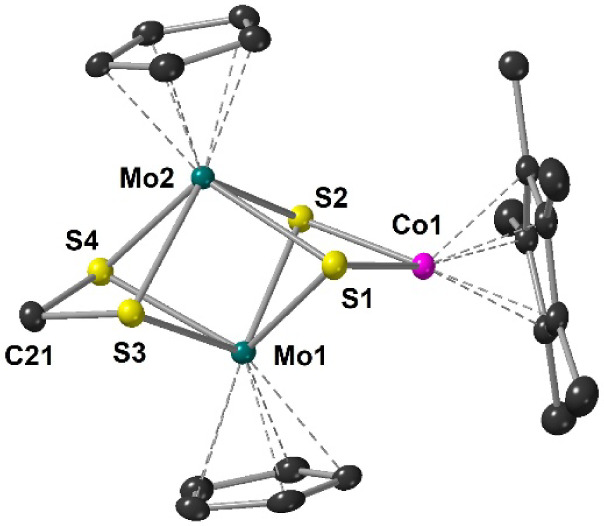
The molecular structure of **1** shown with 50% probability ellipsoids. Hydrogen atoms and cocrystallized solvent molecules have been removed for clarity. Key: C = dark gray ellipsoids, S = yellow ellipsoids, Co = pink ellipsoid, Mo = teal ellipsoids.

**Table 1:** Selected Bond Lengths (Å) of [(CpMo)_2_(Cp*Co)­(μ_3_-S)_2_(μ_2_-S_2_CH_2_)] **(1)**, with (Cp*_3_Mo_3_S_4_)­CoCl; A Model for Cobalt Adsorption at the Basal Plane of MoS_2_, Included for Comparison^26^

Bond	1	(Cp*_3_Mo_3_S_4_)CoCl
Co–Mo	2.829(4), 3.456(6)	2.778(6)
Co–S	2.170(7), 2.167(6)	2.215(8), 2.217(7), 2.219(7)
Co–C(Cp*)	2.076 (avg)	–
Mo–Mo	2.626(3)	2.848 (avg)
Mo–S_Co_	2.408(6), 2.417(6)	2.337(7), 2.341(7)
	2.435(6), 2.435(6)	2.334(7), 2.338(7)
		2.336(7), 2.337(8)
Mo–S_Mo_	2.464(6), 2.465(6)	2.342(7), 2.344(7), 2.345(7)
	2.456(6), 2.459(6)	
Mo–C(Cp)	2.324 (avg)	2.368 (avg)
Mo–S–Co	Mo1: 76.15(2), 76.01(2)	–
	Mo2: 97.12(2), 97.30(2)	

The Co–S distances in **1** (Co–S = 2.170(7) and 2.167(6) Å) are comparable to reports of Co­(III) thiolate complexes in the literature, which typically range from 2.11 to 2.21 Å.^47–50^ For example, the mononuclear, tetrahedral Co­(III) complex [Cp*Co­(bdt)] (bdt = 1,2-benzenedithiolate) has Co–S distances of 2.133(1) Å. This observation suggests that the Co­(II) starting material undergoes oxidation upon coordination to the dimer assembly, consistent with electron transfer from the cobalt center to the thiomolybdate framework. Cobalt oxidation is further supported by shorter Co–C­(Cp*) distances in **1** (2.076(2) Å) compared to Cp*_3_CoMo_2_S_4_ (2.328(3) Å). The assignment of a Co­(III) center in **1** necessitates two Mo­(III) centers to balance the charge of the complex, providing an overall oxidation state distribution of Co­(III)­Mo­(III)_2_. Inspection of the Mo–S bond distances supports the presence of Mo­(III) centers. The Mo–S distances (Mo–S_Co_ (avg) = 2.412 Å and 2.435 Å, and Mo–S_Mo_ (avg) = 2.457 Å and 2.464 Å), both asymmetric due to the structural distortion described above, are consistent with reports of Mo­(III) sulfide complexes in the literature.^30,51–54^ For example, the Mo­(III) sulfide complex [(Cp’Mo)_2_(μ_2_-SCH_3_)_2_(μ_2_-S_2_CH_2_)] (Cp’ = CH_3_C_5_H_4_) possesses Mo–S bond distances of 2.448(1) Å and 2.453(1) Å.^30^

Comparison of the solid-state structure of **1** with that of (Cp*_3_Mo_3_S_4_)­CoCl,^26^ a molecular model of cobalt adsorption at the basal plane of MoS_2_, provides insight into the structural consequences of cobalt incorporation at the edge versus the basal plane of MoS_2_. Reports of Co-doped MoS_2_ materials typically invoke Co­(II) and Mo­(IV) oxidation states for cobalt incorporated within the basal plane, whereas the electronic structure of cobalt adsorption at the edge sites is less understood.^55,56^ In this context, we note that the proposed oxidation state distribution in (Cp*_3_Mo_3_S_4_)­CoCl, Co­(II)­Mo­(III)_2_Mo­(IV), differs from that assigned for **1** but is more consistent with electronic structure descriptions of Co-MoS_2_. The difference in cobalt oxidation state in the two model complexes (e.g., (Cp*_3_Mo_3_S_4_)­CoCl and **1**) is reflected in the Co–S bond distances of the two complexes, where longer bonds are found in (Cp*_3_Mo_3_S_4_)­CoCl (Co–S (avg) = 2.217 Å), consistent with a more reduced cobalt center in the basal plane model. Bond metric analysis suggests that the Mo centers in **1** are reduced (Mo­(III)_2_), reflected in the short Mo–Mo bond distance of 2.6258(2) Å. Comparatively, the average Mo–Mo distances in (Cp*_3_Mo_3_S_4_)­CoCl are substantially longer (Mo–Mo = 2.838(6) Å), consistent with the more electron-deficient nature of the basal plane of MoS_2_ compared to the edge sites. Taken together, the structural metrics indicate strong electronic communication between cobalt and the thiomolybdate framework, whereas in the basal-plane model, (Cp*_3_Mo_3_S_4_)­CoCl, the cobalt atom seems to be electronically decoupled from the molybdenum centers. This is consistent with the differing electronic distributions of the two assemblies, where cobalt oxidation in **1** is accompanied by the reduction of the molybdenum centers, while more localized structural changes occur in (Cp*_3_Mo_3_S_4_)­CoCl, like those reported for Co-MoS_2_. These observations suggest that cobalt uptake at MoS_2_ edge sites induces more pronounced structural changes compared to the basal plane.

The electronic properties of **1** were further interrogated by electron paramagnetic resonance (EPR) and electronic absorption spectroscopies. The EPR spectrum of **1** was recorded in a frozen toluene solution at 10 K with an X-band spectrometer. The spectrum shows a very weak, broad signal at a value of g ∼ 2; spin quantification suggests this signal is produced by <0.05% of the sample (Figure S17), indicating that the observed signal results from a minor paramagnetic impurity. The weak EPR signal and the well-resolved, sharp resonances observed in the ^1^H NMR spectrum of **1** that are located close to their diamagnetic reference values (Figure S2) suggest that **1** is diamagnetic. This was further confirmed by an Evans method measurement (μ_eff_ = 0). We credit the observed diamagnetic electron structure to strongly antiferromagnetically coupled electrons of the two Mo­(III) centers in **1**.^57–59^ The electronic absorption spectrum of **1** was collected in DMF and compared to that of [(CpMo)_2_(μ_2_-S)_2_(μ_2_-S_2_CH_2_)]. The electronic absorption spectrum of **1** exhibits three major features: an intense absorption in the UV region at 340 nm (ε = 29,519 M^–1^ cm^–1^), a band in the visible region at 614 nm (5,742 M^–1^ cm^–1^), and a broad feature in the near-infrared (NIR) region centered at 955 nm (2,513 M^–1^ cm^–1^) (Figure 3). The spectrum of the free thiomolybdate dimer in comparison is quite different; it possesses two main absorption features positioned at 610 nm (ε = 1,316 M^–1^ cm^–1^) and 724 nm (ε = 1,429 M^–1^ cm^–1^) (Figure S12). These transitions are attributed to ligand-to-metal charge transfer (LMCT) transitions from the Cp π-orbitals to the d-orbitals of Mo.

**Figure 3:**
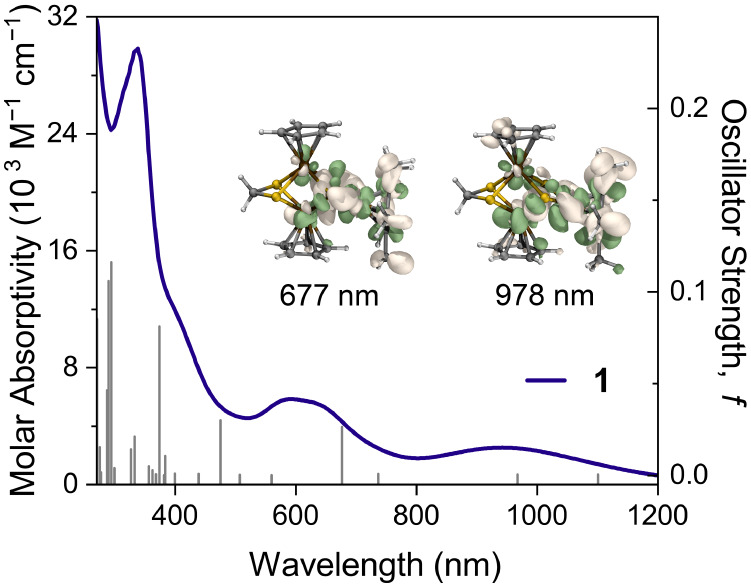
Experimentally recorded and calculated electronic absorption spectrum of **1** collected at room temperature in DMF. The right vertical axes represent the calculated oscillator strength in light gray sticks. The inset figures represent the electron density difference (EDD) maps of selected transitions (electrons move from white regions → green regions upon excitation) with an isovalue of 0.0007 a.u.

To elucidate the nature of the electronic excitations, electron density difference (EDD) maps were generated from TD-DFT results and are presented in Figure 3. The lowest-energy transitions extending beyond the visible region (λ > 900 nm) originate from a combination of LMCT (π­(Cp) →d­(Mo) and π­(Cp*)→d­(Co)), metal-centered d–d transitions (d­(Mo/Co) →d­(Co)), and π–π* excitations localized on the Cp* ligand. The mid energy absorption band between 500–700 nm arises predominantly from metal–to–metal charge transfer (MMCT, d­(Mo)→d­(Co)), coupled with LMCT contributions from p­(μ_3_-S) to d­(Co), with minor participation from Cp*-based π–π* transitions. The high-energy band centered at 340 nm is dominated by the MMCT processes involving d­(Mo)/d­(Mo–Mo)→d­(Co)), accompanied by intermetal d­(Mo)→d­(Mo’) excitations. Taken together, the combined experimental and computational results highlight Mo–Co electronic communication and support the assigned Co­(III)­Mo­(III)_2_ electronic configuration of **1**.

To probe the electronic effects arising from cobalt coordination to the thiomolybdate dimer, cyclic voltammetry of **1** was performed and compared to that of [(CpMo)_2_(μ_2_-S)_2_(μ_2_-S_2_CH_2_)] (Figure 4 and Table S4). The cyclic voltammogram (CV) of [(CpMo)_2_(μ_2_-S)_2_(μ_2_-S_2_CH_2_)] displays two reversible redox events with *E*_1/2_ values of −1.72 V ([(CpMo)_2_(μ_2_-S)_2_(μ_2_-S_2_CH_2_)]^0/–^) and −0.14 V ([(CpMo)_2_(μ_2_-S)_2_(μ_2_-S_2_CH_2_)]^0/+^) vs Fc^+/0^ in DMF (0.1 M TBAPF_6_ as the supporting electrolyte). Comparatively, the CV of **1** under identical experimental conditions exhibits three reversible redox events with *E*_1/2_ values of −1.97, −1.04, and −0.49 V (vs Fc^+/0^). We note that the incorporation of cobalt results in access to an additional redox couple, indicative of significant perturbations to the electronic structure of **1** compared to the dimeric species.

**Figure 4:**
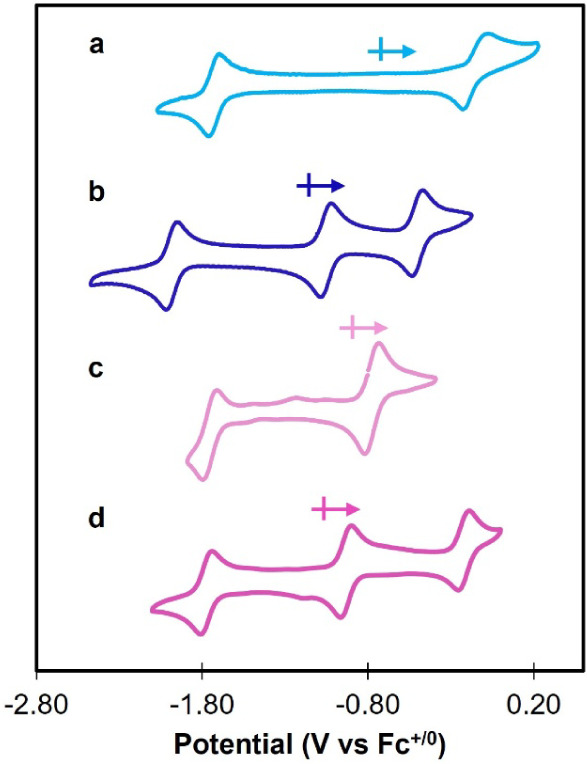
Cyclic voltammograms of (a) [(CpMo)_2_(μ_2_-S)_2_(μ_2_-S_2_CH_2_)], (b) **1**, (c) (Cp*_3_Mo_3_S_4_)­CoCl, and (d) Cp*_3_CoMo_2_S_4_. Data were acquired in DMF for (a), (b), and (d) and in DCM for (c), with 0.1 M supporting electrolyte (TBAPF_6_) at a scan rate of 200 mV s^–1^. Potential values are expressed with reference to the ferrocenium/ferrocene redox couple (Fc^+/0^).

With the formal oxidation state distribution of Co­(III)­Mo­(III)_2_ in **1**, the reductive event is assigned to the Co­(III)/(II) redox couple, while the two oxidative events are attributed to oxidations of the thiomolybdate assembly (Mo­(III)_2_/Mo­(IV)_2_). Nevertheless, the DFT frontier orbital analyses indicate that the redox events are not fully localized at a single metal site. In particular, the lowest unoccupied molecular orbital (LUMO) is distributed over the CoMo_2_S_4_ core with substantial cobalt contribution but clear participation of the adjacent Mo–S framework, consistent with a predominant Co reduction rather than a purely metal-centered process (Figure S5). By contrast, the highest occupied molecular orbital (HOMO) is also delocalized across the cluster and contains significant Mo character, supporting the assignment of the oxidative events as electron removal from the Mo_2_S_4_ assembly (Figure S5). Taken together, these results suggest that the reduction and oxidation events are best described as predominantly Co- and thiomolybdate-weighted, respectively, while retaining substantial covalency and electronic delocalization across the entire CoMo_2_S_4_ framework.

Though the redox potentials of Co­(III)/Co­(II) couples can vary, the structurally similar dinuclear Cp*Co dithiolene complex, [Cp*Co­(btt)­CoCp*] (btt = 1,2,4,5-benzenetetrathiolate), possesses two reversible Co­(III)/Co­(II) redox events at *E*_1/2_ = −1.63 and −1.37 V (vs Fc^+/0^) in dichloromethane, similar to those measured for **1**.^47^ Comparison of the Mo-weighted redox events to those of the related thiomolybdate dimer [(CpMo)_2_(μ_2_-S)_2_(μ_2_-S_2_CH_2_)] reveals a much larger separation between the two Mo-based redox events (Δ*E*_1/2_ = 1.58 V), corresponding to an exceptionally large comproportionation constant (*K*_c_ = 6.02 × 10^26^) and, therefore, a highly stabilized mixed-valent electronic structure. In **1**, by contrast, the analogous redox separation is much smaller (Δ*E*_1/2_ = 0.55 V), resulting in a dramatic reduction, but still of significance, in the comproportionation constant (*K*_c_ = 2.10 × 10^9^). Thus, although the thiomolybdate core in **1** remains electronically coupled, coordination of cobalt clearly attenuates the extent of Mo–Mo electronic delocalization relative to the parent dimer. This decrease is consistent with the DFT description of the frontier orbitals, in which the electronic structure is redistributed over the broader CoMo_2_S_4_ framework rather than being concentrated within the Mo_2_ unit alone; accordingly, incorporation of cobalt appears to divert electron density into a more extensively mixed, but less strongly Mo–Mo-delocalized, manifold.

Comparison of the CV of **1** with that of (Cp*_3_Mo_3_S_4_)­CoCl reveals distinct differences in their electronic properties (Figure 4). The CV of (Cp*_3_Mo_3_S_4_)­CoCl possesses a reversible oxidation at −0.77 V (vs Fc^+/0^) and a reversible reduction at −1.75 V (vs Fc^+/0^) (DCM, TBAPF_6_). Based on the proposed oxidation state distribution of Co­(II)­Mo­(III)_2_Mo­(IV) in (Cp*_3_Mo_3_S_4_)­CoCl, the oxidative feature is assigned to a Mo­(III)/Mo­(IV) event, while the reductive feature corresponds to the Mo­(IV)/Mo­(III) redox couple. Notably, no cobalt-based redox processes are observed in the electrochemical window of (Cp*_3_Mo_3_S_4_)­CoCl, in contrast to **1,** where an accessible Co­(III)/Co­(II) reduction is present. These differences suggest that cobalt adsorbed to the trisulfide face of the Mo_3_S_4_ assembly may be electronically decoupled from the molybdenum centers, highlighting how cobalt incorporation in a dimeric (“Mo_2_S_4_”) versus trimeric (“Mo_3_S_4_”) molybdenum framework can modulate the electronic structures of the resulting complexes. Comparison of the CV of **1** with Cp*_3_CoMo_2_S_4_, which serves as a model of *substitutional* cobalt doping at the basal plane of MoS_2_ (rather than cobalt adsorption to the surface), further supports this hypothesis. Despite differences in structure and formal oxidation state assignments (Co­(II)­Mo­(V)­Mo­(IV) for Cp*_3_CoMo_2_S_4_), the electrochemical profiles of the two disparate heterometallic assemblies are similar. The CV of Cp*_3_CoMo_2_S_4_ exhibits three reversible redox events, with a reductive event located at *E*_1/2_ = −1.80 V and two oxidative events at *E*_1/2_ = −0.96 V and −0.25 V, all of which are shifted anodically relative to that of **1**.^29^ These processes are assigned to a Mo­(V)/Mo­(IV) reduction, and Mo­(IV)/Mo­(V) and Co­(II)/Co­(III) oxidation events. The similarities in the cyclic voltammograms of **1** and Cp*_3_CoMo_2_S_4_ suggest that cobalt incorporation into the disparate thiomolybdate frameworks has a similar influence on the redox properties of the complexes, despite the distinct geometries and coordination environments associated with each compound.

#### Isolation of Redoxomers of [(CpMo)_2_(Cp*Co)­(μ_3_-S)_2_(μ_2_-S_2_CH_2_)]

To interrogate how changes in the redox state of the complex modulate the electronic structure of **1**, we next aimed to isolate its reduced and oxidized congeners, namely [(CpMo)_2_(μ_2_-S)_2_(μ_2_-S_2_CH_2_)]^1–^ (**2**) and [(CpMo)_2_(μ_2_-S)_2_(μ_2_-S_2_CH_2_)]^1+^ (**3**). The reversibility of the Co­(III)/Co­(II) and Mo­(III)/Mo­(IV) redox couples observed in cyclic voltammetry experiments suggests that the reduced and oxidized redoxmers of **1** would be isolable, motivating subsequent chronoamperometry experiments. Controlled potential electrolysis of **1** at −2.2 V in DMF (with 0.1 M TBAPF_6_ as the supporting electrolyte) results in a color change of the solution from dark blue-green to brown-green, consistent with the formation of a new species in solution (Figures S6–S8). Prior to electrolysis, the open-circuit potential (OCP) of **1** was observed at −0.932 V (vs Fc^+/0^). After bulk reduction, the OCP shifts to −1.79 V (vs Fc^+/0^). This value is lower than the potential of the target Co­(III)/Co­(II) redox couple, indicating successful reduction of **1**. Notably, the CV obtained after bulk electrolysis is identical to that of **1**, apart from the shifted OCP value, indicating that reduction does not result in decomposition of the complex under the conditions of bulk electrolysis experiments. The electronic absorption spectrum of the bulk-electrolyzed solution possesses a set of broad transitions, with band positions similar to that of **1** (λ = 340 nm (ε = 12,134 M^–1^ cm^–1^), 606 nm (ε = 1,031 M^–1^ cm^–1^) and 948 (ε = 281 M^–1^ cm^–1^)) (Figure 5). The primary difference between the spectra of **1** and its reduced derivative is a pronounced decrease in the molar absorptivity of the transitions. Collectively, these observations support the successful one-electron electrochemical reduction of **1** to form the reduced species in solution.

**Figure 5:**
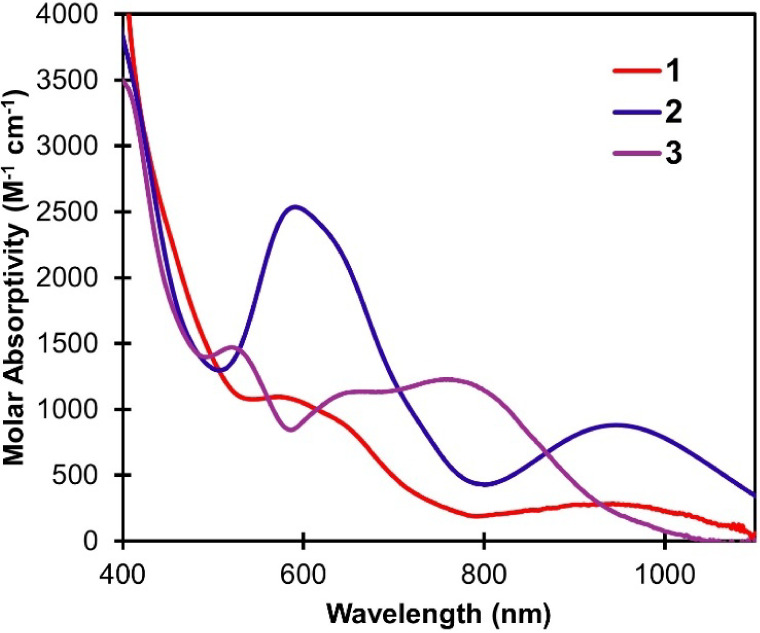
Electronic absorption spectra for [(CpMo)_2_(Cp*Co)­(μ_3_-S)_2_(μ_2_-S_2_CH_2_)] (**1**), [K­(2.2.2-cryptand)]­[Cp_2_Mo_2_(SCH_2_S)­S_2_Cp*Co] (**2**) and [Cp_2_Mo_2_(SCH_2_S)­S_2_Cp*Co]­[PF_6_] (**3**) collected at room temperature in DMF.

Encouraged by the results from bulk electrolysis, chemical reduction of **1** was next pursued. Treatment of a THF solution of **1** with KC_8_ and 2.2.2-cryptand results in the formation of the target reduced complex, [K­(2.2.2-cryptand)]­[(CpMo)_2_(Cp*Co)­(μ_3_-S)_2_(μ_2_-S_2_CH_2_)] (**2**), in excellent yield (Scheme 2). Following workup (see Experimental section for details), **2** was isolated as a dark green-brown solid. Characterization of **2** via ^1^H NMR spectroscopy reveals paramagnetically broadened and shifted resonances at δ = 5.60 and 2.65 ppm, corresponding to the CpMo (10H) and Cp*Co protons (15H), respectively (Figure S3). A set of resonances at δ = 3.67 ppm, overlapping with the deuterated solvent signal, was assigned to 2.2.2-cryptand and the methanedithiolate ligand.

**Scheme 2:**
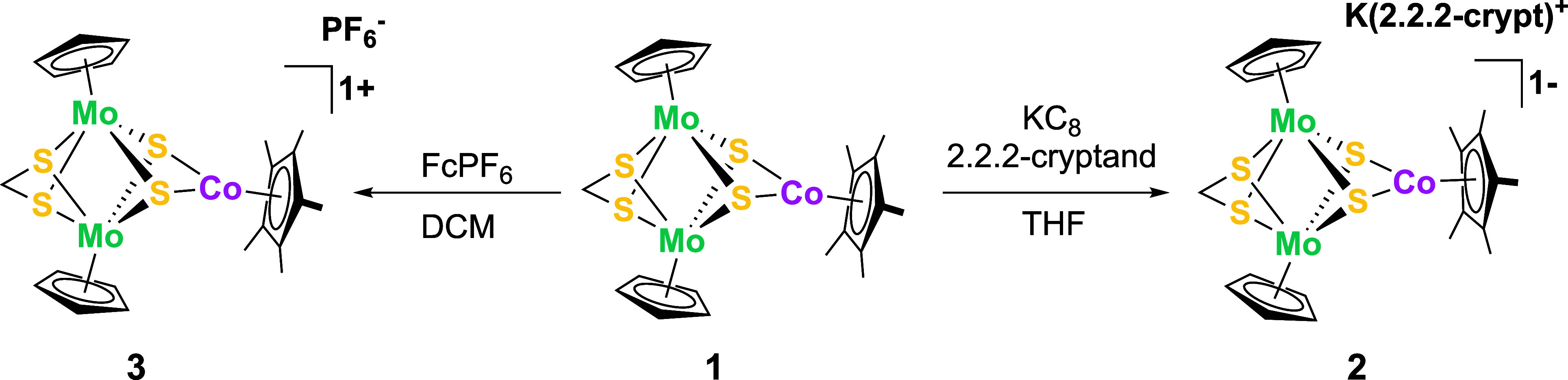
Syntheses of [K­(2.2.2-Cryptand)]­[(CpMo)_2_(Cp*Co)­(μ_3_-S)_2_(μ_2_-S_2_CH_2_)] (**2**, Right) and [(CpMo)_2_(Cp*Co)­(μ_3_-S)_2_(μ_2_-S_2_CH_2_)]­[PF_6_] (**3**, Left)

To probe the electronic consequences of Co-centered reduction, complex **2** was characterized by electronic absorption and EPR spectroscopies. The electronic absorption spectrum of **2** closely matches that obtained following bulk electrolysis, indicating that the same reduced species is formed under both electrochemical and chemical conditions (Figure S13). TD-DFT calculations of **2** reveal that the lower-energy band corresponds to a Co d–d transition, and the mid-energy absorption band (500–700 nm) originates from a LMCT feature arising from π­(Cp*) to Co (Figure S14). The higher-energy transition is assigned to metal-centered absorption, accompanied by minor contributions from metal–metal’–to–ligand charge transfer (MM’LCT), where a Mo–Mo bond forms and electrons flow from the metal–metal bond to the capped Cp ligands. The X-band EPR spectrum of **2,** recorded in a frozen 2-MeTHF solution, exhibits an anisotropic eight-line hyperfine pattern for a cobalt-based species with a *g*-value centered at 2.03 (*S* = 1/2, *I* = 7/2) (Figures 6 and S17). For example, the EPR spectrum of the Co­(II) starting material, (Cp*CoCl)_2_, shows a rhombic signal with three components split due to hyperfine coupling.^32^ However, the lines are substantially broadened, consistent with additional unresolved interactions involving the adjacent Mo centers.^32,60–63^ Such broadening reflects partial spin delocalization onto Mo-based orbitals and/or hyperfine coupling to active Mo atoms. By means of the Evans method, the effective magnetic moment (μ_eff_) of **2** in solution at room temperature was determined to be 1.75 μ_B_, consistent with the presence of a low-spin, paramagnetic Co­(II) complex with one unpaired electron. Together, these observations confirm that the reduction of **1** to **2** is predominantly localized at the cobalt center.

**Figure 6:**
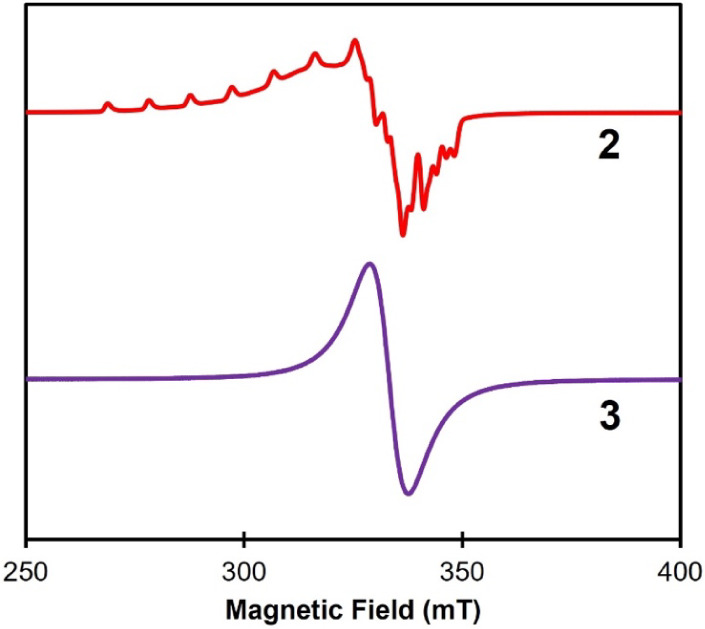
X-band EPR spectra of **2** and **3** recorded at 10 K in 2-MeTHF and MeCN, respectively.

Crystals of **2** suitable for SCXRD analysis were grown via vapor diffusion of pentane into a saturated solution of the product in THF at −30 °C. Refinement of the data confirms the structural composition of **2** as the anticipated anionic “CoMo_2_S_4_” species, with a cationic K (2.2.2-cryptand) counterion present to balance the charge (Figure 7, Tables 2 and S2). The Co–S bond lengths of the reduced product (Co–S = 2.1913(13) Å) are slightly lengthened from that of **1** (Co–S = 2.1681 Å), consistent with reduction localized at Co in **2**. Inspection of the Co–C­(Cp*) distances further supports this hypothesis; in line with the larger ionic radius of the Co­(II) ion,^64^ the Co–C­(Cp*) bonds are longer in **2** (avg. = 2.091 Å) compared to **1** (avg. = 2.076 Å). It is also important to note that minor changes to the Mo_2_S_4_ core are observed upon reduction. The Mo–Mo distances are similar in both redoxmers (Mo–Mo = 2.615(6) Å (**2**) and 2.626(3) Å (**1**)), and minor differences are found in the Mo–S_Mo_ bond lengths (2.456(1) Å (**2**) vs 2.461(6) Å (**1**)).

**Figure 7:**
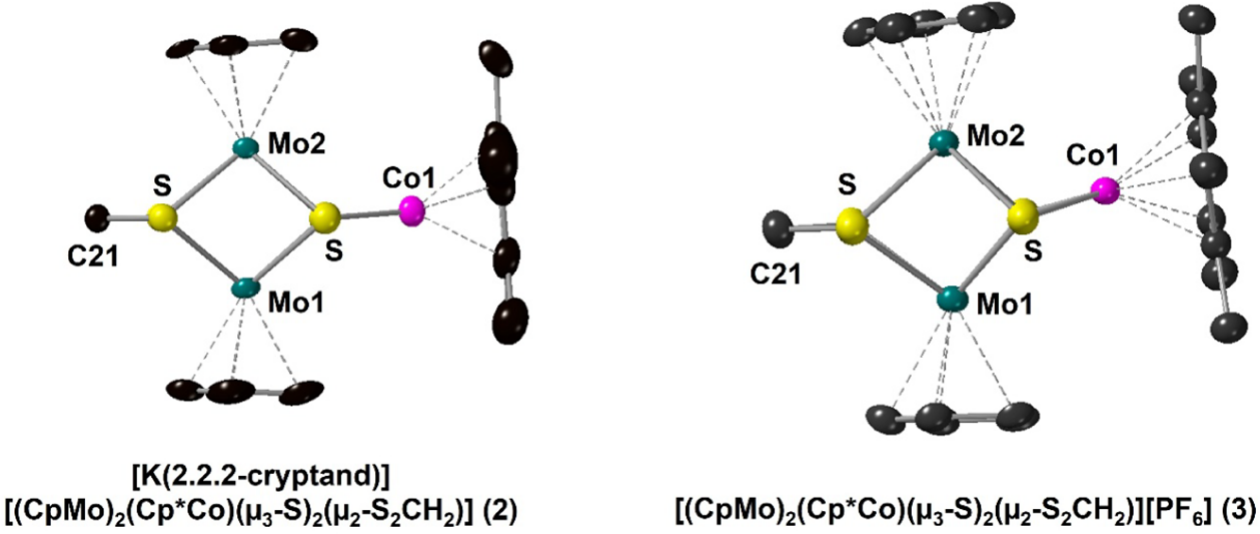
Molecular structures of **2** and **3** are shown with 50% probability ellipsoids. Hydrogen atoms and cocrystallized solvent molecules have been removed for clarity. Key: C = black ellipsoids, S = yellow ellipsoids, Co = pink ellipsoid, Mo = teal ellipsoids.

**Table 2:** Selected Bond Lengths (Å) and Angles of [(CpMo)_2_(Cp*Co)­(μ_3_-S)_2_(μ_2_-S_2_CH_2_)] **(1)**, [K­(2.2.2-cryptand)]­[Cp_2_Mo_2_(SCH_2_S)­S_2_Cp*Co] (**2**), and [Cp_2_Mo_2_(SCH_2_S)­S_2_Cp*Co]­[PF_6_] (**3**)

Bond	1 Co(III)Mo(III)_2_	2 Co(II)Mo(III)_2_	3 Co(III)Mo(IV)Mo(III)
Co–Mo	2.829(4), 3.456(6)	3.421(1), 3.325(1)	2.800(4), 3.113(6)
Co–S	2.170(7), 2.167(6)	2.191(1)	2.139(7), 2.143(8)
Co–C(Cp*)	2.076	2.091	2.103
Mo–Mo	2.626(3)	2.615(6)	2.595(3)
Mo–S_Co_	2.408(6), 2.417(6)	2.461(1), 2.459(1)	2.397(6), 2.400(7)
	2.435(6), 2.435(6)		2.410(7), 2.419(7)
Mo–S_Mo_	2.464(6), 2.465(6)	2.447(1), 2.457(1)	2.456(7), 2.461(7)
	2.456(6), 2.459(6)		2.460(8), 2.467(7)
Mo–C(Cp)	2.324	2.362	2.330
Mo–S–Co	Mo1: 76.15(2), 76.01(2)	–	Mo1: 75.93 (3)
	Mo2: 97.12(2), 97.30(2)		Mo2: 85.90(3), 86.05(3)

With an understanding of the electronic structure of **2**, we next targeted the isolation of the oxidized complex. TD-DFT calculations showed that the highest occupied molecular orbital of **1** possesses substantial Mo–S character, suggesting that oxidation would occur within the thiomolybdate core rather than at cobalt. Controlled potential electrolysis of **1** at −0.75 V in DMF (0.1 M TBAPF_6_) results in a rapid color change of the solution to brown and a shift in the OCP from −0.921 V (vs Fc^+/0^) (before electrolysis) to −0.566 V (vs Fc^+/0^) (after electrolysis) (Figures S9 and S10). This value is more positive than the potential of the targeted redox couple, indicating successful bulk oxidation of **1** under the applied conditions. The electronic absorption spectrum of the bulk oxidized solution reveals a set of broad transitions, with discernible maxima at λ = 400 nm (ε = 3,031 M^–1^ cm^–1^), 529 nm (ε = 1,257 M^–1^ cm^–1^), 664 nm (ε = 984 M^–1^ cm^–1^), and 780 nm (ε = 1,047 M^–1^ cm^–1^) (Figure 5). The calculated TD-DFT spectrum of **3** shows good agreement with the experimental data, revealing that the lowest-energy transition (calculated wavelength of 798 nm) is dominated by Co-centered character (Figure S15), while the middle-energy absorption arises from a combination of metal- and ligand-based charge transfer. The higher-energy transition (calculated wavelength of 412 nm) is assigned to ligand-to-ligand and ligand-to-metal charge transfer events. The differential absorption features observed across the redox series illustrate the influence of the formal oxidation states of the metal center(s) on electronic structure, highlighting the role of electronic communication among the metal centers in this system.

To chemically access the oxidized species, **1** was treated with one equivalent of ferrocenium hexafluorophosphate (FcPF_6_) in DCM, resulting in an immediate color change to dark brown. Following workup (see Experimental section for details), **3** was characterized by ^1^H NMR spectroscopy (Figure S4). The spectrum features a single paramagnetically broadened resonance at 8.15 ppm, in contrast to the sharp, well-resolved signals observed for the closed-shell, diamagnetic complex **1**. While signals corresponding to the Cp* protons on cobalt, Cp protons on molybdenum, and the methylene protons of the tether might be expected, the presence of one broad resonance serves as an initial indication that oxidation perturbs the electronic structure of the complex, either through electron delocalization or rapid relaxation on the NMR time scale, leading to fast proton exchange and the appearance of a single broad resonance. By means of the Evans method, the effective magnetic moment (μ_eff_) of **3** was determined to be 3.85 μ_B_, consistent with the presence of multiple unpaired electrons and partial antiferromagnetic coupling between the Mo­(III) and Mo­(IV) centers, with a diamagnetic Co­(III) center. The relatively large magnetic moment is inconsistent with a fully localized *S* = 1/2 system, but instead supports a spin-coupled, mixed-valent electronic structure where electron density is delocalized across the molybdenum centers while containing significant magnetic interactions.

Complex **3** was further characterized by electronic absorption and EPR spectroscopies. The electronic absorption spectrum of **3** closely matches that of the bulk-electrolyzed compound, indicating that the same species is generated upon chemical oxidation (Figure S16). The X-band EPR spectrum of **3**, recorded in a frozen acetonitrile solution, exhibits a broad signal at a g value of 2.01 (Figures 6 and S17), consistent with a paramagnetic species. Notably, no evidence of hyperfine coupling to the^95^ Mo (15.72%; *I* = 5/2) or ^97^Mo (9.46%; *I* = 5/2) nuclei is observed. This value is in close agreement with that reported for the mixed-valent molybdenum dimer, [CpMo­(SCH_3_)_2_]_2_[PF_6_] (*g* = 2.009), for which hyperfine splitting is also unresolved.^65^ The absence of hyperfine splitting, coupled with the broadness of the signal, is in good agreement with the magnetic data for **3**. This suggests significant delocalization of the unpaired electrons and magnetic coupling across the multimetallic core, indicative of strong metal–metal communication in the oxidized complex.

To investigate changes in the solid-state structure upon oxidation, single crystals of **3** suitable for X-ray analysis were grown from the slow diffusion of Et_2_O into a concentrated DCM solution of the product at −30 °C (Figure 7, Tables 2 and S3). Refinement of the data reveals the anticipated cationic species, with a PF_6_^–^ counterion present to balance the charge. Notably, the asymmetric Co–Mo interaction observed in **1** is retained in the solid-state structure of **3**, in which the cobalt center is tilted toward Mo(1). The Co–Mo(1) and Co–Mo(2) distances (2.800(4) and 3.113(6) Å, respectively) are contracted compared to those in **1**, consistent with oxidation of the complex. However, all other bond metrics of the complex exhibit minimal changes. The average Mo–S_Mo_ distances remain unchanged (2.461 Å for **1** and **3**) and there is a minor difference in the Mo–S_Co_ distances (**1** = 2.424 Å; **3** = 2.407 Å). The Co–S bond lengths in **3** (2.139(7) Å and 2.143(8) Å) are slightly shorter than those in **1** (Co–S = 2.170(7) and 2.167(6) Å), indicating increased interaction between the cobalt center and the thiomolybdate framework upon oxidation. We attribute this observation to the smaller size of the oxidized molybdenum center, which translates to the cobalt atom being pulled closer to the thiomolybdate framework. Taken together, these structural metrics suggest that oxidation enhances the metal–metal interactions within the complex without significant structural changes occurring at a single molybdenum center, supporting the description of **3** as a delocalized, mixed-valent system where the Mo centers likely share spin density.

Recently, our research team reported Cp*_3_CoMo_2_S_4_ as an electrocatalyst for proton reduction, modeling the basal plane of Co-doped MoS_2_. Our results reveal that incorporation of the Co heterometal into the thiomolybdate assembly improves the catalytic performance of the cluster relative to its homometallic congener, Cp*_3_Mo_3_S_4_.^29^ Since the parent thiomolybdate assembly [(CpMo)_2_(μ_2_-S)_2_(μ_2_-S_2_CH_2_)] was originally utilized as a proton reduction electrocatalyst,^27,30^ we became interested in how Co incorporation at the edge site in **1** influences catalytic reactivity. The electrocatalytic activities of 1 and [(CpMo)_2_(μ_2_-S)_2_(μ_2_-S_2_CH_2_)] (0.5 mM) were examined with HNEt_3_BF_4_ (30 mM) in DMF at −1.75 V over a 90-min controlled-potential electrolysis (CPE) experiment (Figure S11). Unfortunately, the parent [(CpMo)_2_(μ_2_-S)_2_(μ_2_-S_2_CH_2_)] accumulated a sustained greater total charge and produced more H_2_ in comparison to **1**, suggesting that incorporation of the Cp*Co fragment attenuates proton reduction reactivity and inhibits the catalytically active site. Thus, whereas cobalt incorporation can be beneficial in extended MoS_2_ materials when it activates comparatively inert basal-plane environments, cobalt installation at the edge-site mimic in **1** appears instead to compromise the native thiomolybdate HER-active motif, thereby attenuating proton reduction catalysis.

## Conclusions

In summary, we report the synthesis and characterization of a cobalt-substituted thiomolybdate dimer that serves as a molecular model for the edge sites of MoS_2_, allowing for the investigation of how cobalt incorporation influences the electronic structure of the thiomolybdate framework. The electronic structure of [(CpMo)_2_(Cp*Co)­(μ_3_-S)_2_(μ_2_-S_2_CH_2_)] (**1**) possesses a closed-shell, diamagnetic Co­(III)­Mo­(III)_2_ ground state, as supported by NMR spectroscopy, the Evans method, and EPR measurements. Electronic absorption spectroscopy, in combination with TD-DFT calculations, reveals several transitions across the UV–vis-NIR regions, including LMCT, MMCT, d–d, and ligand-centered π–π* transitions, consistent with extensive electronic communication in **1**. Electrochemical studies reveal that **1** possesses three accessible redox processes corresponding to a Co­(III)/Co­(II) reduction and Mo-centered oxidation events.

We find that modulation of the redox state induces pronounced changes in electron structure across the series: the closed-shell Co­(III)­Mo­(III)_2_ ground state of **1** can undergo cobalt-centered reduction to generate a Co­(II)­Mo­(III)_2_ species in **2**, while one-electron oxidation affords a delocalized, mixed-valent system in **3**. The findings herein provide molecular-level understanding into how Co-adsorption at MoS_2_ edge sites modulates electronic structure and highlight the importance of metal–metal communication in these systems, offering insights into the electronic effects of transition metal uptake at these sites. We believe this understanding will prove helpful for future investigations into how catalytic HER will be impacted in these molecular models.

## Supplementary Material



## Notes

(PDF) The Supporting Information is available free of charge at https://pubs.acs.org/doi/10.1021/acs.inorgchem.6c01985.

Additional experimental procedures; spectroscopic and crystallographic data for complexes **1**, **2**, and **3** (CCDC 2547365–2547367); and computational details (PDF)
