# Supplementary material for: Electronic Properties of a Structural Model of Single-Atom Co-Adsorption to MoS 2 Edge Sites
Source: Inorg Chem. 2026 Jun 3;65(23):13265–75. doi: 10.1021/acs.inorgchem.6c01985 (PMC13273804; doi:10.1021/acs.inorgchem.6c01985)
Supplement: Supplementary file 1 [file ic6c01985_si_001.pdf]

Electronic Supporting Information

Electronic Properties of a Structural Model of Single-atom Co-Adsorption to MoS_2 Edge Sites

Leyla R. Valerio[‡], Isabella Florez Monroy[‡], Zhou Lu*, William W. Brennessel, and Ellen M. Matson*

Department of Chemistry, University of Rochester, Rochester NY 14627, USA

[‡]Authors contributed equally to this work

Corresponding Author E-mail Addresses:

Zhou Lu: zhoulu@rochester.edu

Ellen M. Matson: matson@chem.rochester.edu

Table of Contents

1. ^{1}H NMR Spectroscopy

Figure S1. ^{1}H NMR spectrum of **1** in C_6D_6……3
Figure S2. Stacked ^{1}H NMR spectra of $[(CpMo)_2(\mu_2\text{-}S)_2(\mu_2\text{-}S_2CH_2)]$ and **1** in C_6D_6……4
Figure S3. ^{1}H NMR spectrum of **2** in THF-d_8……5
Figure S4. ^{1}H NMR spectrum of **3** in CD_3CN……6

2. Single Crystal X-ray Diffraction

Table S1. Crystallographic parameters for **1**……7
Table S2. Crystallographic parameters for **2**……8
Table S3. Crystallographic parameters for **3**……9

3. Electrochemistry

Table S4. Redox potentials for $[(CpMo)_2(\mu_2\text{-}S)_2(\mu_2\text{-}S_2CH_2)]$, **1**, $(Cp^*_3Mo_3S_4)CoCl$, and $Cp^*_3CoMo_2S_4$……10
Figure S5. Frontier molecular orbital counters of **1** with the isovalue of 0.02 a.u……10
Figure S6. Pre bulk electrolysis CV of **1** (1 mM) in DMF (0.1 M $TBAPF_6$). Scan rate = 200 mV/s……11
Figure S7. Bulk reduction of a 1 mM solution of **1** in DMF (0.1 M $TBAPF_6$). Chronoamperometry was performed at -2.2 V vs $Fc^{+/0}$……12
Figure S8. Post bulk electrolysis (reduction) CV of **1** (1 mM) in DMF (0.1 M $TBAPF_6$). Scan rate = 200 mV/s……13
Figure S9. Bulk oxidation of a 1 mM solution of **1** in DMF (0.1 M $TBAPF_6$). Chronoamperometry was performed at -0.75 V vs $Fc^{+/0}$……14
Figure S10. Post bulk electrolysis (oxidation) CV of **1** (1 mM) in DMF (0.1 M $TBAPF_6$). Scan rate = 200 mV/s……15
Figure S11. Controlled potential electrolysis experiments of $[(CpMo)_2(\mu_2\text{-}S)_2(\mu_2\text{-}S_2CH_2)]$ and **1** in the presence of 30 mM of $HNEt_3BF_4$……15

4. Electronic Absorption Spectroscopy

Figure S12. Electronic absorption spectrum of $[(CpMo)_2(\mu_2\text{-}S)_2(\mu_2\text{-}S_2CH_2)]$……16
Figure S13. Electronic absorption spectrum of **2** in THF……17
Figure S14. Experimentally recorded and calculated electronic absorption spectra of **2** collected at room temperature in DMF……18
Figure S15. Experimentally recorded and calculated electronic absorption spectra of **3** collected at room temperature in DMF……19
Figure S16. Electronic absorption spectrum of **3** in MeCN……20

5. EPR Spectroscopy

Figure S17. EPR spectrum of **1** recorded in a frozen toluene solution……21

6. Computational Details

Coordinates of DFT-optimized Structures……22

1. ^{1}H NMR Spectroscopy

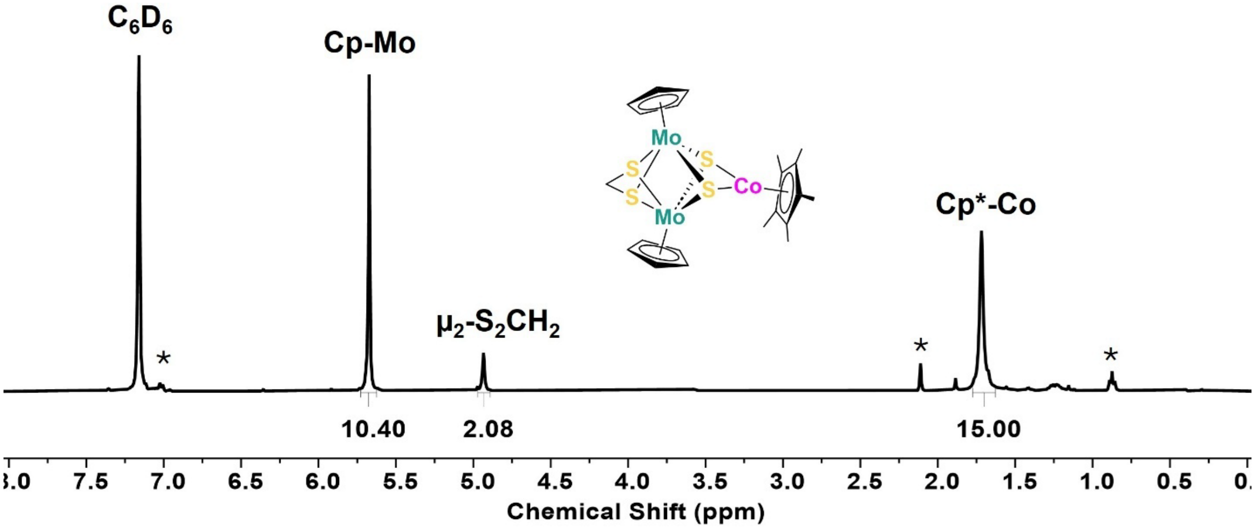

Figure S1. ^{1}H NMR spectrum of **1** in C_6D_6. Peaks labelled with (*) correspond to residual solvent in the sample.

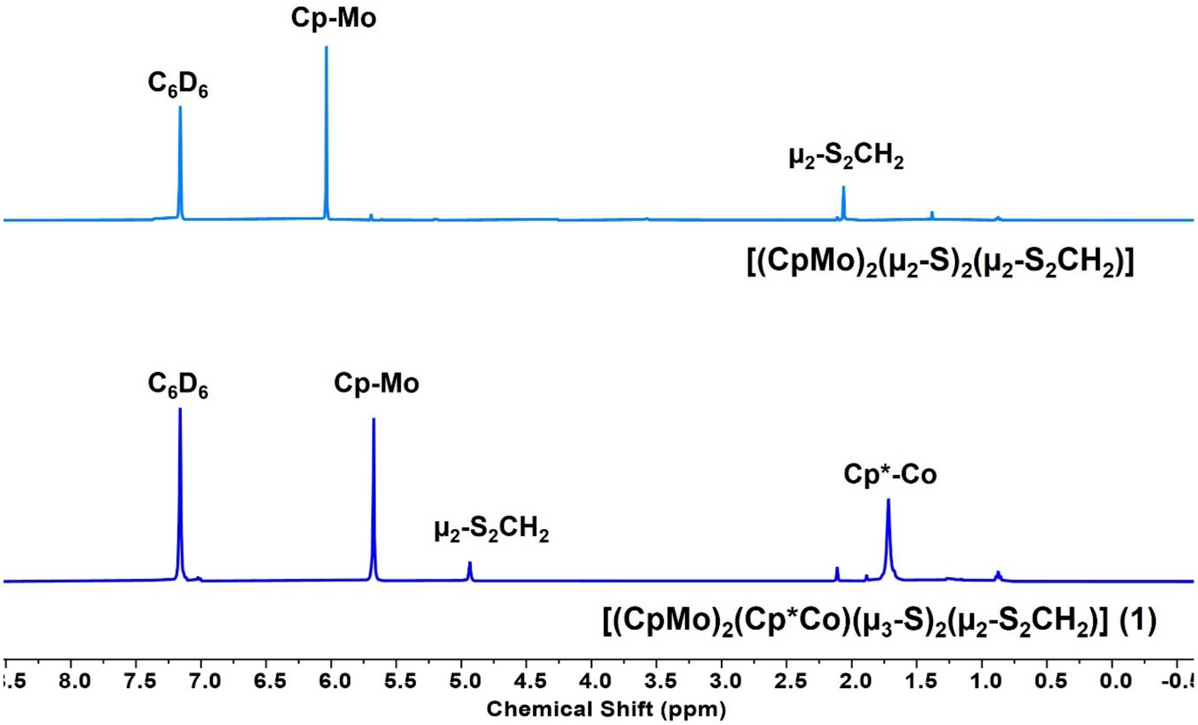

Figure S2. Stacked ^{1}H NMR spectra of [(CpMo)$_2$(μ_2-S)$_2$(μ_2-S_2CH_2)] and **1** in C_6D_6.

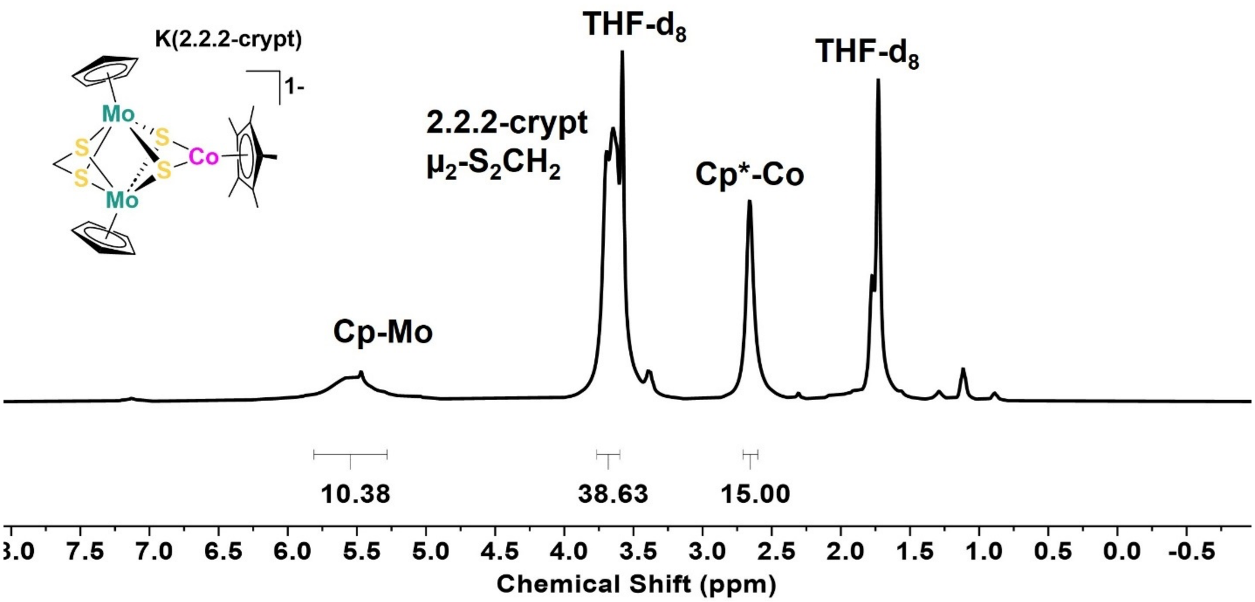

Figure S3. ^{1}H NMR spectrum of **2** in THF-d_8.

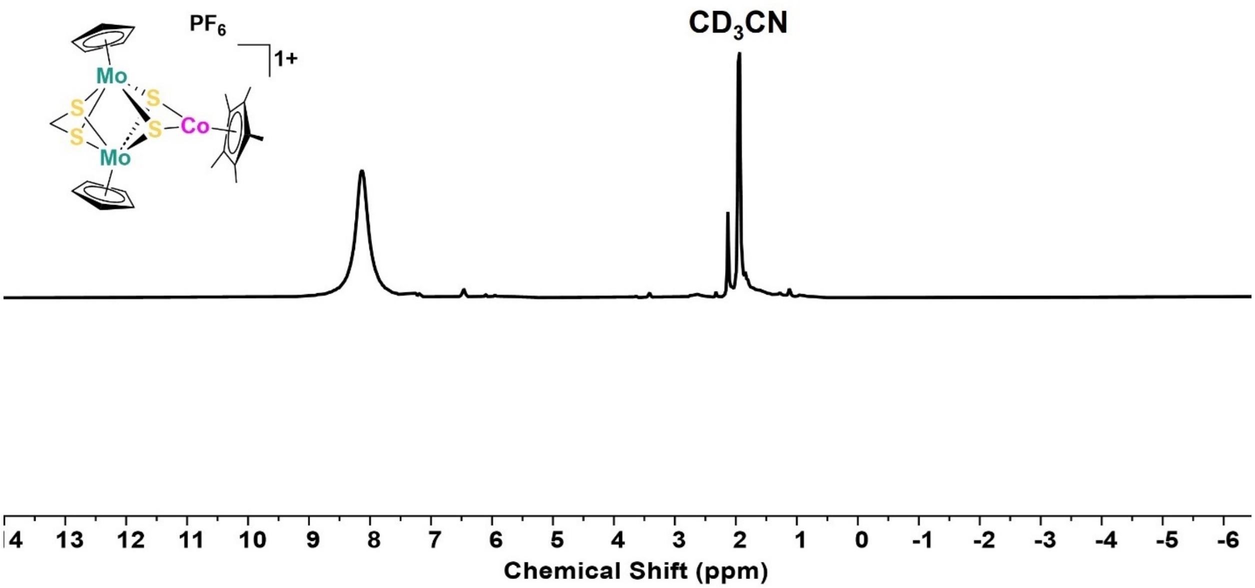

Figure S4. ^{1}H NMR spectrum of **3** in CD_3CN.

2. Single Crystal X-ray Diffraction

Table S1. Crystallographic parameters for **1**

**Empirical Formula**	$C_{21}H_{27}CoMo_2S_4$
**Formula Weight**	658.47
**Temperature**	100.00(10) K
**Wavelength**	CuKα (λ = 1.54184)
**Crystal System**	monoclinic
**Space Group**	$P2_1/n$
**Unit cell Dimensions**	a = 8.1539010)<br>b = 10.86950(10)<br>c = 25.3430(2)<br>α = 90°<br>β = 91.0710(10)°<br>γ = 90°
**Volume/Å^3**	2245.73(4)
**Z**	4
**Reflections Collected**	36660
**Independent Reflections**	4829
**Goodness-of-Fit on F^2**	1.037
**Final R indices [I>2sigma(I)]**	R_1 = 0.0214, wR_2 = 0.0475

Table S2. Crystallographic parameters for **2**

**Empirical Formula**	$C_{47}H_{79}CoKMo_2N_2O_8S_4$
**Formula Weight**	1218.27
**Temperature**	100.00(10) K
**Wavelength**	CuKα (λ = 1.54184)
**Crystal System**	orthorhombic
**Space Group**	*Pnma*
**Unit cell Dimensions**	a = 13.68910(10)<br>b = 9.98570(10)<br>c = 38.6452(3)<br>α = 90°<br>β = 90°<br>γ = 90°
**Volume/Å^3**	5282.61(8)
**Z**	4
**Reflections Collected**	48311
**Independent Reflections**	6004
**Goodness-of-Fit on F^2**	1.057
**Final R indices [I>2sigma(I)]**	R_1 = 0.0487, wR_2 = 0.1199

Table S3. Crystallographic parameters for **3**

**Empirical Formula**	$C_{22}H_{29}Cl_2CoF_6Mo_2PS_4$
**Formula Weight**	888.37
**Temperature**	172.99(10) K
**Wavelength**	CuKα (λ = 1.54184)
**Crystal System**	triclinic
**Space Group**	*P*-1
**Unit cell Dimensions**	a = 10.9701(2)<br>b = 11.2098(2)<br>c = 14.2455(2)<br>α = 110.9770(10)°<br>β = 101.374(10)°<br>γ = 106.353(2)°
**Volume/Å^3**	1480.17(5)
**Z**	2
**Reflections Collected**	48944
**Independent Reflections**	6334
**Goodness-of-Fit on F^2**	1.063
**Final R indices [I>2sigma(I)]**	R_1 = 0.0298, wR_2 = 0.0793

3. Electrochemistry

Table S4. Redox potentials for [$(CpMo)_2(\mu_2\text{-}S)_2(\mu_2\text{-}S_2CH_2)$], **1**, $(Cp^*{}_3Mo_3S_4)CoCl$, and $Cp^*{}_3CoMo_2S_4$

Redox Couple	[$(CpMo)_2(S)_2(S_2CH_2)$]	1	$(Cp^*{}_3Mo_3S_4)CoCl$	$Cp^*{}_3CoMo_2S_4$
**1+/2+**	–	-0.49	–	-0.25
**0/1+**	-0.14	-1.04	-0.77	-0.96
**0/1-**	-1.72	-1.97	-1.75	-1.80

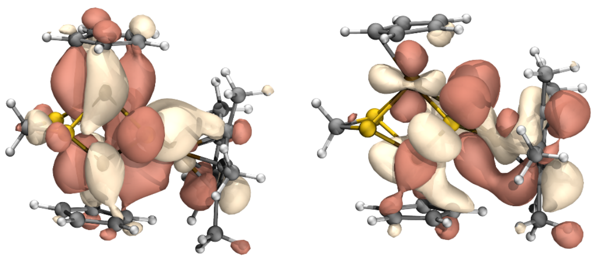

Figure S5. Frontier molecular orbital counters of **1** with the isovalue of 0.02 a.u.

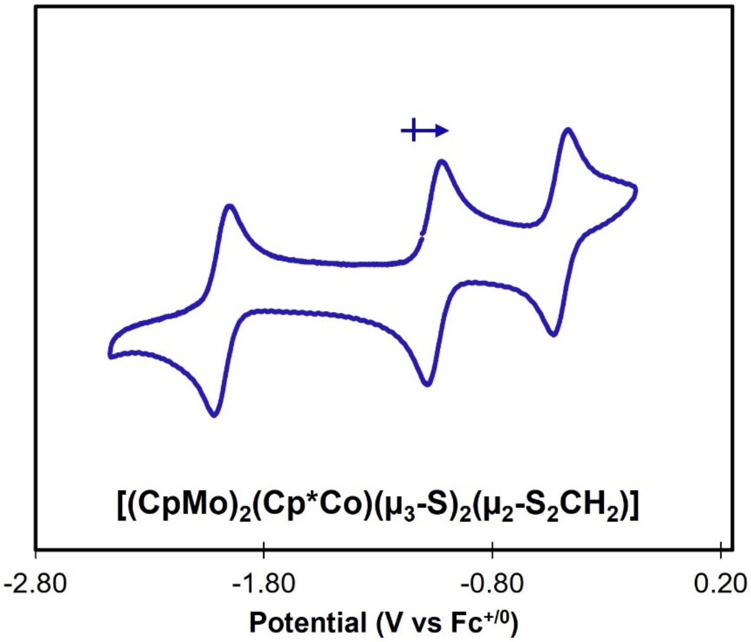

Figure S6. Pre bulk electrolysis CV of **1** (1 mM) in DMF (0.1 M $TBAPF_6$). Scan rate = 200 mV/s.

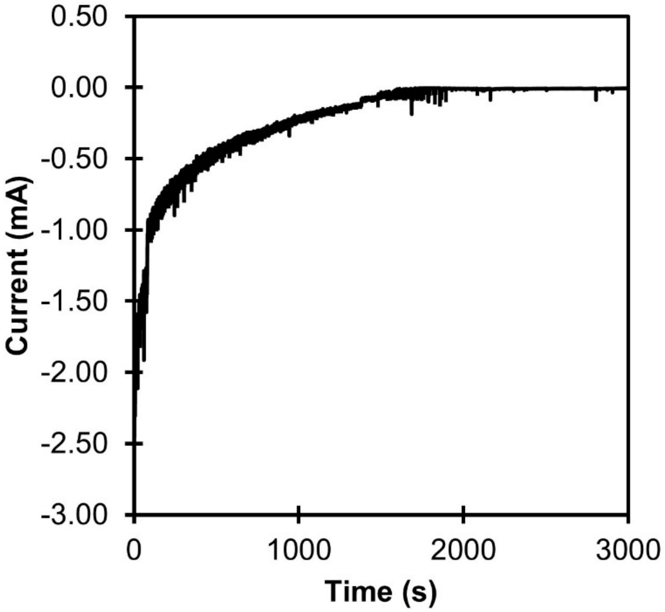

Figure S7. Bulk reduction of a 1 mM solution of **1** in DMF (0.1 M $TBAPF_6$). Chronoamperometry was performed at -2.2 V vs $Fc^{+/0}$

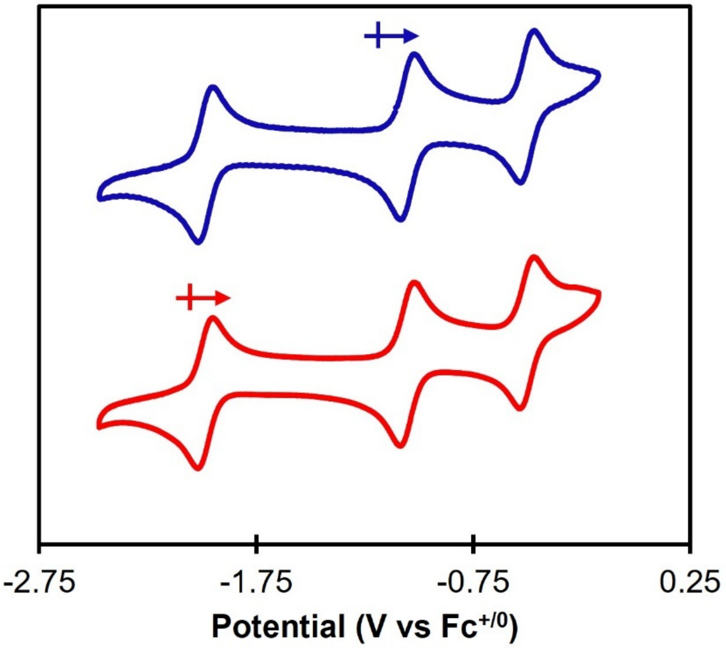

Figure S8. Post bulk electrolysis (reduction) CV of **1** (1 mM) in DMF (0.1 M $TBAPF_6$). Scan rate = 200 mV/s.

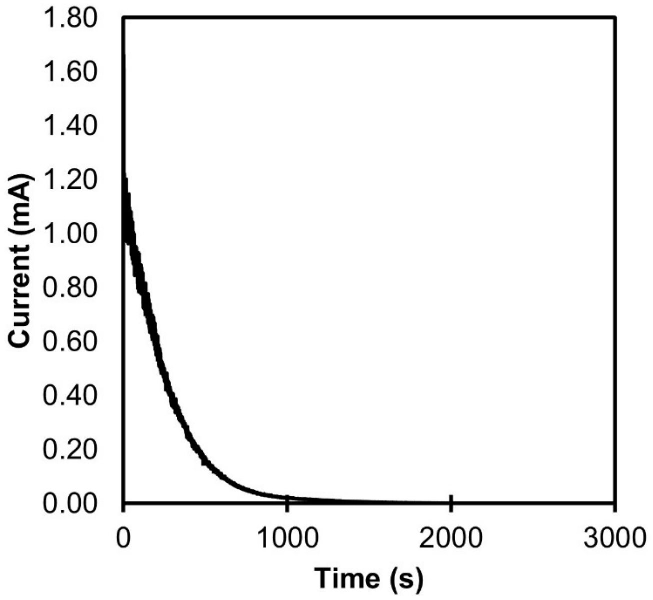

Figure S9. Bulk oxidation of a 1 mM solution of **1** in DMF (0.1 M $TBAPF_6$). Chronoamperometry was performed at -0.75 V vs $Fc^{+/0}$.

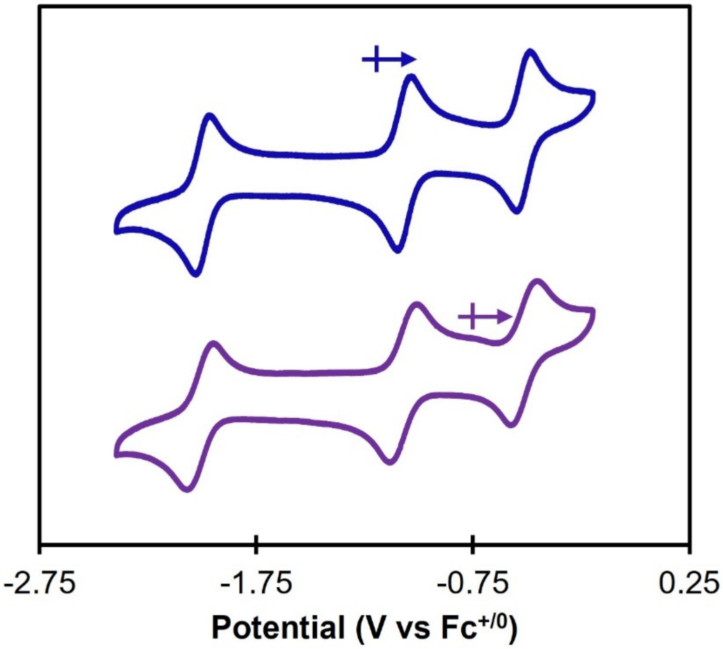

Figure S10. Post bulk electrolysis (oxidation) CV of **1** (1 mM) in DMF (0.1 M $TBAPF_6$). Scan rate = 200 mV/s.

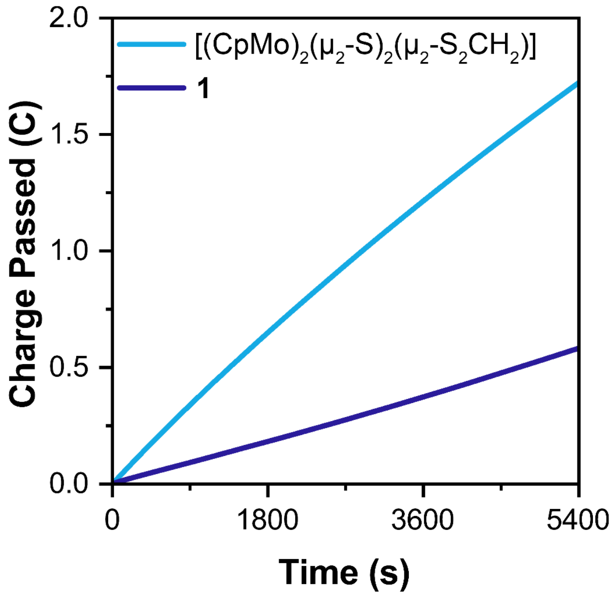

Figure S11. Accumulation of charge versus time in the controlled potential electrolysis of a 0.5 mM DMF solution of [$(CpMo)_2(\mu_2\text{-}S)_2(\mu_2\text{-}S_2CH_2)$] and **1** in the presence of 30 mM of $HNEt_3BF_4$ over 90 minutes at -1.75 V. The working electrode is a glassy carbon rod of thickness of 3 mm and length immersed in solution is ~1 cm; the reference and counter electrodes are an Ag/$AgNO_3$ pseudo reference electrode and Pt wire, respectively.

4. Electronic Absorption Spectroscopy

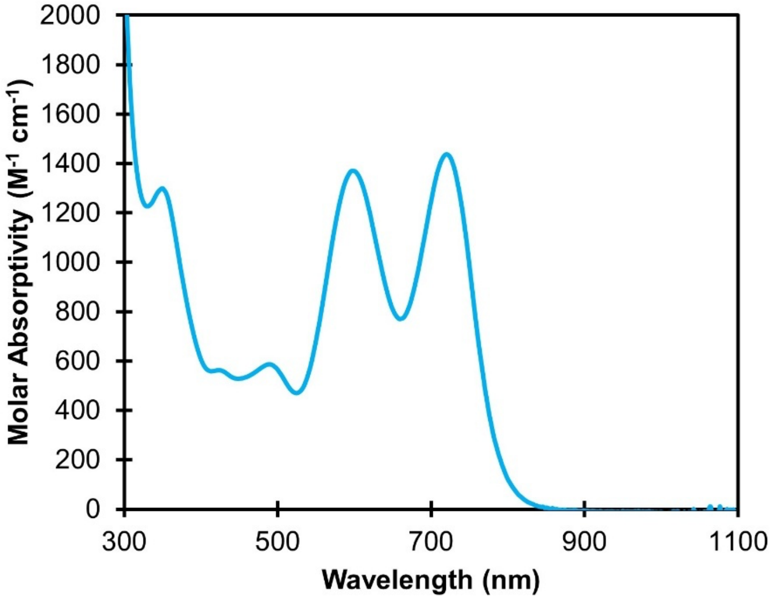

Figure S12. Electronic absorption spectrum of $[(CpMo)_2(\mu_2\text{-}S)_2(\mu_2\text{-}S_2CH_2)]$ in DCM.

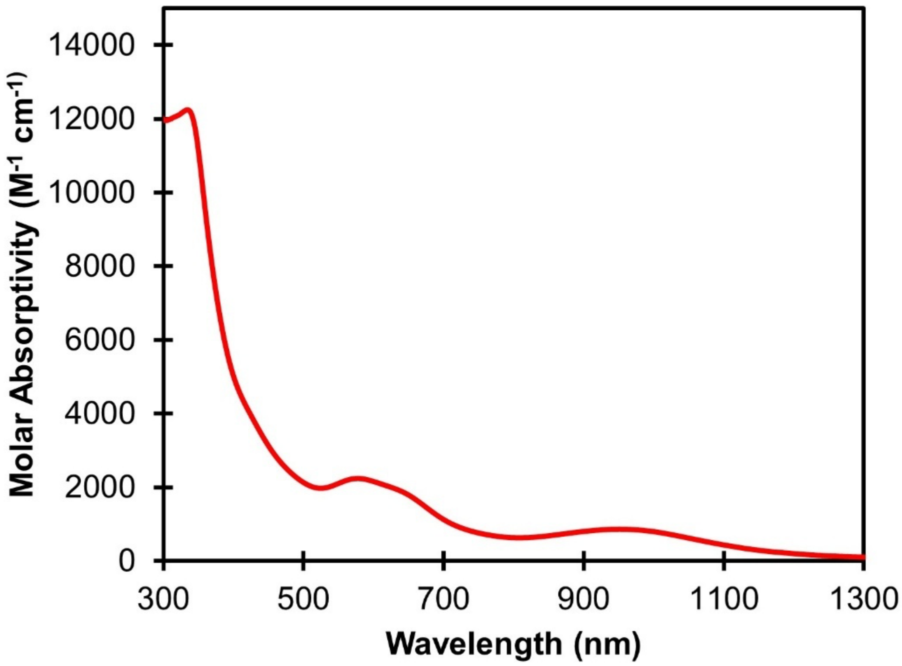

Figure S13. Electronic absorption spectrum of **2** in THF.

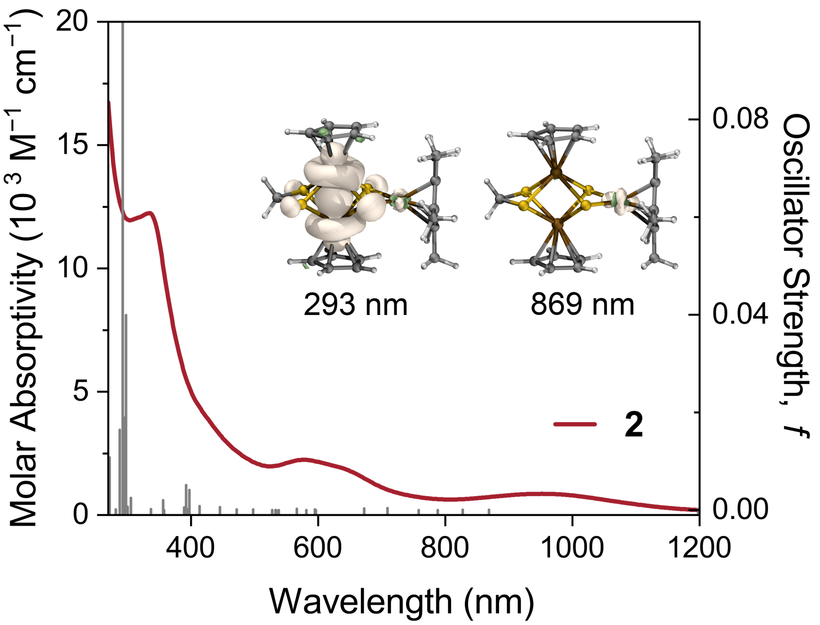

Figure S14. Experimentally recorded and calculated electronic absorption spectra of **2** collected at room temperature in DMF.

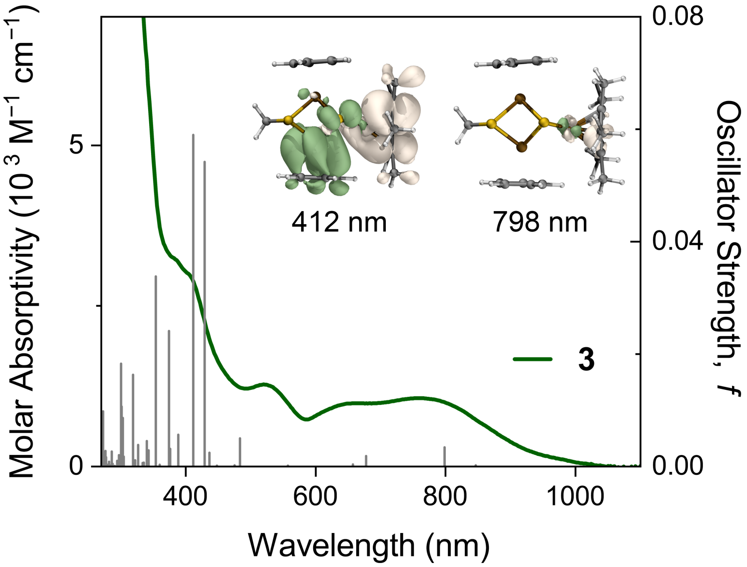

Figure S15. Experimentally recorded and calculated electronic absorption spectra of **3** collected at room temperature in DMF.

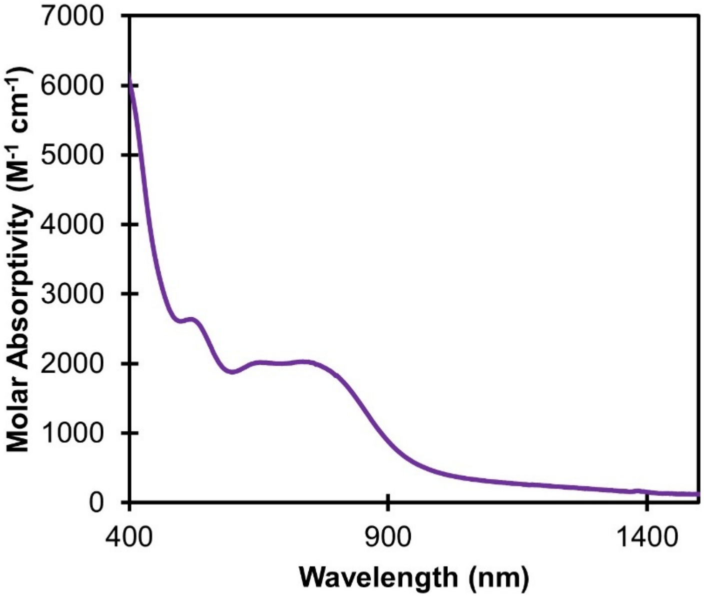

Figure S16. Electronic absorption spectrum of **3** in MeCN.

5. EPR Spectroscopy

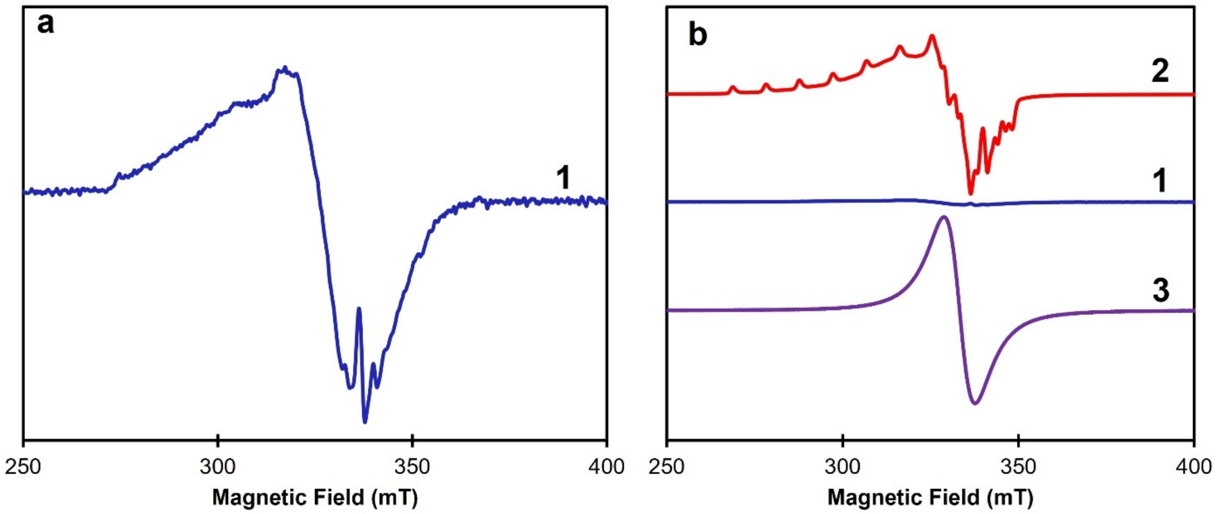

Figure S17. (**a**) EPR spectrum of **1** in frozen toluene solution at 10K. (**b**) Stacked EPR spectra of **1** (toluene), **2** (2-MeTHF), and **3** (MeCN) recorded in frozen solution at 10K.

6. Computational Details

Coordinates

1

Mo	2.10162751	5.60119502	15.90037803
Mo	3.84794029	6.41775867	17.67122454
Co	2.46031229	8.20160941	15.03517268
S	4.09189750	6.80953981	15.28247319
S	1.88135513	7.71444486	17.06009346
S	3.96038274	4.18658917	16.65719810
S	1.93414662	5.00896471	18.28095894
C	3.45471814	9.57887494	13.93207707
C	2.75951234	10.18863961	15.02349901
C	1.35339536	9.92790991	14.83539327
C	1.19185524	9.18747294	13.63080604
C	2.48886167	8.93692691	13.08392012
C	4.92605565	9.63558781	13.68015238
H	5.48327277	9.78230566	14.60621896
H	5.16265175	10.46889197	13.00866879
H	5.28603904	8.71611994	13.21689178
C	3.36644816	10.99637086	16.12394446
H	3.41142116	12.05791395	15.85308479
H	4.38269316	10.66378880	16.34072923
H	2.78308835	10.90922529	17.04168553
C	0.25096250	10.40764792	15.72160463
H	0.60399391	9.72989093	15.69471299
H	0.09617043	11.39785879	15.40507769
H	0.57946937	10.48056390	16.75897150
C	0.12372820	8.82719801	13.01978885
H	0.04073901	8.01016793	12.30157350
H	0.53442617	9.68687124	12.47827427
H	0.85698235	8.54141679	13.77631522
C	2.80991901	8.21220324	11.81566423
H	3.70407564	7.59639564	11.93232939
H	2.99331546	8.91404985	10.99417182
H	1.99304086	7.55776410	11.50442314
C	0.96339315	5.73292186	13.89783465
H	1.08995461	6.54717492	13.21142168
C	0.03645951	5.63473456	14.91401508
H	0.75520117	6.39464433	15.17508206
C	0.09794336	4.37689417	15.53904595
H	0.48664243	4.02492699	16.37497695
C	1.19551044	3.70087081	14.95067906

H	1.55554057	2.72072842	15.22099526
C	1.73682959	4.53504657	13.92132959
H	2.55375602	4.28869431	13.26329213
C	5.69918573	7.80311147	18.10979542
H	6.15375164	8.40124318	17.33528448
C	4.65307203	8.21867239	18.96267613
H	4.17597586	9.18616235	18.94622974
C	4.28007958	7.12509093	19.78852957
H	3.51423735	7.14021917	20.54746136
C	5.11569784	6.00414260	19.44139020
H	5.15004472	5.05315086	19.94883560
C	5.99844698	6.44183749	18.38948627
H	6.76264926	5.85214583	17.90914650
C	2.90018097	3.48298694	17.97502837
H	2.28135775	2.65335388	17.62512625
H	3.47802103	3.18250139	18.85034712

2

Mo	1.98318341	5.23301951	15.91357015
Mo	3.76932412	6.29203124	17.50668427
Co	2.48174048	8.37797664	15.07880015
S	3.82232212	6.64231966	15.06927465
S	1.68353760	7.47281903	16.91867460
S	3.99644928	4.01095433	16.61267018
S	2.01683723	4.77431248	18.32982920
C	3.52265722	9.83990429	14.04335658
C	2.78268036	10.44986881	15.08902470
C	1.39646608	10.12050541	14.88542375
C	1.27871353	9.37096930	13.66879517
C	2.59316863	9.16384481	13.16636656
C	5.00609544	9.86019154	13.85885830
H	5.51345085	10.16486990	14.77702177
H	5.30607387	10.55875316	13.06673000
H	5.37829685	8.86753437	13.59242908
C	3.32779515	11.25495599	16.22461591
H	3.30433163	12.33276857	16.01185902
H	4.36382400	10.98473515	16.44196270
H	2.74754454	11.08850633	17.13541044
C	0.26730678	10.54290600	15.76928533
H	0.57544492	9.85331712	15.68797463

	-		
H	0.09357799	11.54757380	15.51205365
H	0.57268724	10.55222173	16.81783434
C	0.00364075	8.89798952	13.04808978
H	0.14817338	7.96893526	12.49172762
H	0.40640972	9.63907299	12.34809321
H	0.76066221	8.71105157	13.80599098
C	2.97607821	8.40918713	11.93333229
H	3.86036861	7.79197291	12.11142237
H	3.19952413	9.08456473	11.09671430
H	2.17100944	7.74434249	11.61129961
C	1.01528658	5.14793352	13.77194476
H	1.39021899	5.78284546	12.98483726
C	0.01757412	5.48230071	14.67247827
H	0.55389767	6.41765412	14.68912648
C	0.13693643	4.44587029	15.63457390
H	0.83639504	4.42538051	16.45522575
C	0.84313911	3.43356593	15.32196531
H	0.93961314	2.46991062	15.79744945
C	1.56331288	3.88928329	14.15334527
H	2.35800855	3.36600002	13.64627512
C	5.53131505	7.83297768	17.75668631
H	5.86773873	8.41552081	16.91304821
C	4.51790348	8.21137412	18.65973714
H	3.95223984	9.12889770	18.61511205
C	4.27307158	7.13001606	19.55037034
H	3.55081138	7.12482419	20.35129677
C	5.16753271	6.05101197	19.19699263
H	5.31527742	5.13608868	19.74969268
C	5.94420885	6.50455068	18.06403731
H	6.71284252	5.94848268	17.55138966
C	3.11184868	3.33022359	18.06486532
H	2.55274466	2.41968705	17.82614269
H	3.78024601	3.15503856	18.91371904

3

Mo	1.97495538	5.53635082	15.84609317
Mo	3.74198370	6.50751809	17.46839900
Co	2.51905328	8.14429184	15.14809213
S	3.98008263	6.58591317	15.06907239
S	1.63891155	7.60589804	17.02017048

S	3.89064778	4.20458301	16.61976648
S	1.91956082	5.05577789	18.25853779
C	3.57157000	9.54927162	14.09604461
C	2.83899716	10.16960433	15.14650262
C	1.44137270	9.90539660	14.91022458
C	1.31416665	9.17929162	13.68441354
C	2.62166615	8.91246587	13.20266007
C	5.04621421	9.62096331	13.87491185
H	5.58437428	9.79967133	14.80608273
H	5.27776824	10.44905998	13.19690605
H	5.43264258	8.70575737	13.42585167
C	3.39544008	10.99721106	16.25716561
H	3.40746825	12.05493964	15.97406503
H	4.42035981	10.71011032	16.49410800
H	2.79311578	10.90612722	17.16181372
C	0.30475689	10.42270836	15.72765208
H	0.55174649	9.74836383	15.69741458
H	0.02045419	11.39161076	15.33374414
H	0.58810152	10.56512785	16.77025618
C	0.01630106	8.84811080	13.02303731
H	0.14945434	8.17456429	12.17590311
H	0.44445076	9.76170209	12.63420507
H	0.69802715	8.39847046	13.71591866
C	2.98914442	8.18478931	11.95131700
H	3.89484524	7.59243885	12.08995085
H	3.17844333	8.89478325	11.13980284
H	2.19370541	7.51475400	11.62291598
C	0.70738819	5.61481816	13.91522862
H	0.75434814	6.44591108	13.23615642
C	0.20476331	5.46989240	15.00288973
H	0.95049275	6.18523979	15.31114507
C	0.03304300	4.21039802	15.60522069
H	0.48635655	3.81635979	16.46521897
C	1.10479738	3.58596724	14.92019659
H	1.52525412	2.61809904	15.14544204
C	1.52701210	4.45163055	13.86817648
H	2.30858813	4.24890553	13.15370267
C	5.53557265	7.97366049	17.84889363
H	5.91903803	8.61292726	17.06980639
C	4.52166682	8.31337286	18.78235269
H	3.99381556	9.25257575	18.82965066
C	4.29629482	7.19559573	19.62055218
H	3.57733271	7.14348514	20.42316568

C	5.16685176	6.14501768	19.19802510
H	5.26946959	5.17804316	19.66513112
C	5.94486741	6.63782665	18.10184948
H	6.70795366	6.09612838	17.56588809
C	2.92970006	3.54876551	18.03052948
H	2.33211744	2.67593466	17.76699093
H	3.55581940	3.33523216	18.89627496
